# Unravelling the clinicopathological and functional significance of replication protein A (RPA) heterotrimeric complex in breast cancers

**DOI:** 10.1038/s41523-023-00524-3

**Published:** 2023-03-30

**Authors:** Mashael Algethami, Michael S. Toss, Corinne L. Woodcock, Chandar Jaipal, Juliette Brownlie, Ahmed Shoqafi, Adel Alblihy, Katia A. Mesquita, Andrew R. Green, Nigel P. Mongan, Jennie N. Jeyapalan, Emad A. Rakha, Srinivasan Madhusudan

**Affiliations:** 1 Nottingham Biodiscovery Institute, School of Medicine, University of Nottingham, University Park, Nottingham, NG7 3RD UK; 2 Department of Pathology, Nottingham University Hospital, City Campus, Hucknall Road, Nottingham, NG51PB UK; 3 Faculty of Medicine and Health Sciences, Centre for Cancer Sciences, University of Nottingham, Sutton Bonington Campus, Sutton Bonington, Leicestershire LE12 5RD UK; 4 Medical Center, King Fahad Security College (KFSC), Riyadh, 11461 Saudi Arabia; 5 Department of Pharmacology, Weill Cornell Medicine, New York, NY 10065 USA; 6 Department of Oncology, Nottingham University Hospitals, Nottingham, NG51PB UK

**Keywords:** Tumour biomarkers, Breast cancer

## Abstract

Replication Protein A (RPA), a heterotrimeric complex consisting of RPA1, 2, and 3 subunits, is a single-stranded DNA (ssDNA)-binding protein that is critically involved in replication, checkpoint regulation and DNA repair. Here we have evaluated RPA in 776 pure ductal carcinomas in situ (DCIS), 239 DCIS that co-exist with invasive breast cancer (IBC), 50 normal breast tissue and 4221 IBC. Transcriptomic [METABRIC cohort (*n* = 1980)] and genomic [TCGA cohort (*n* = 1090)] evaluations were completed. Preclinically, RPA deficient cells were tested for cisplatin sensitivity and Olaparib induced synthetic lethality. Low RPA linked to aggressive DCIS, aggressive IBC, and shorter survival outcomes. At the transcriptomic level, *low RPA* tumours overexpress pseudogene/lncRNA as well as genes involved in chemical carcinogenesis, and drug metabolism. Low *RPA* remains linked with poor outcome. RPA deficient cells are sensitive to cisplatin and Olaparib induced synthetic lethality. We conclude that RPA directed precision oncology strategy is feasible in breast cancers.

## Introduction

Replication Protein A (RPA) is essential for DNA replication, repair, and recombination^1–8^. RPA is a critical single-stranded DNA (ssDNA)-binding protein that coats and protect exposed ssDNA from endogenous nucleases. In addition, RPA provides a platform for recruitment of factors required during DNA damage signalling, DNA repair, and DNA replication. RPA is a multi-domain heterotrimeric protein complex consisting of RPA1 (70 kDa), RPA2 (32 kDa), and RPA3 (14 kDa) sub-units^1–8^. The RPA complex contains six oligonucleotide/oligosaccharide-binding (OB)-fold domains, also termed DNA-binding domains (DBDs). RPA1 subunit has DBD-A, DBD-B, DBD-C and DBD-F domains. RPA2 has DBD-D and RPA3 has DBD-E domain. The DBD-C, DBD-D and DBD-E domains are involved in inter-subunit interactions (trimerization core). The DBD-A, DBD-B, DBD-C and DBD-D domains promote ssDNA binding^1–8^. The N-terminal domain of RPA1 and the C-terminus of RPA2 are involved in protein-protein interactions necessary for DNA replication, repair and recombination^1–8^. Key interactors of RPA include proteins involved in DNA replication (BID, Cdc45, ETTA1, nucleolin, SENP6), DNA repair (53BP1, BRCA2, ATRIP, DNA-PKcs, FANCJ, MRN complex, 9-1-1 complex, XPA, XPG, XPF-ERCC1) and DNA recombination (RAD51, RAD52, DNA2, DSS1, WRN, BLM)^1–8^. Post-translational modification of RPA also facilitates its various functions. This includes phosphorylation in response to genotoxic stress (mediated via ATM, ATR, CDK, or DNA-PKcs enzymes), acetylation in response to UV light (mediated via GCN5 and PCAF proteins), ubiquitylation in response to ssDNA (mediated via RFWD3 and PRP15 proteins) and SUMOylation (mediated via SUMO-2/3) during homologous recombination^1–8^. In addition, RPA has emerging roles in telomere maintenance, transcription, RNA metabolism, cGAS-STING pathway (involved in microbial and viral infections)^1–8^ and retro-transposition^9^. A role for RPA in cancer pathogenesis, prognosis and response to cytotoxic therapy has been described^1–8^. RPA1 missense mutation can promote the development of lymphoid tumours in mice^10^. Biallelic somatic mutation of RPA1 has been described in human pancreatic tumours^11^. Altered expression of RPA protein has been observed in brain, bladder, colon, oesophageal, gastric, and liver cancers^12–20^. However, the role of RPA in breast cancer is largely unknown.

In the current study, we have comprehensively investigated RPA at the genomic, transcriptomic, proteomic level in large clinical cohorts of breast cancer and its precursor (ductal carcinoma in situ (DCIS)). Preclinically, RPA depleted breast cancer cells were investigated for platinum sensitivity and Olaparib directed synthetic lethality.

## Results

We first immunohistochemically profiled RPA1, 2 and 3 in a cohort of 776 pure DCIS, 239 DCIS that co-exist with invasive breast cancer (IBC), 50 normal breast tissue and 4221 IBC. Patient demographics are summarized in Supplementary Tables 1 and 2. Immunohistochemical markers evaluated are summarized in Supplementary Table 3. At the transcriptomic level, *RPA1, 2* and *3* were evaluated in the METABRIC cohort (*n* = 1980). Furthermore, *RPA1, 2* and *3* mutations, copy number changes, epigenetic modifications, and genome wide consequences of low *RPA1, 2 or 3* were evaluated in the TCGA cohort (*n* = 1090).

### RPA deficiency is associated with aggressive breast cancer

#### RPA1

**Fig. 1:**
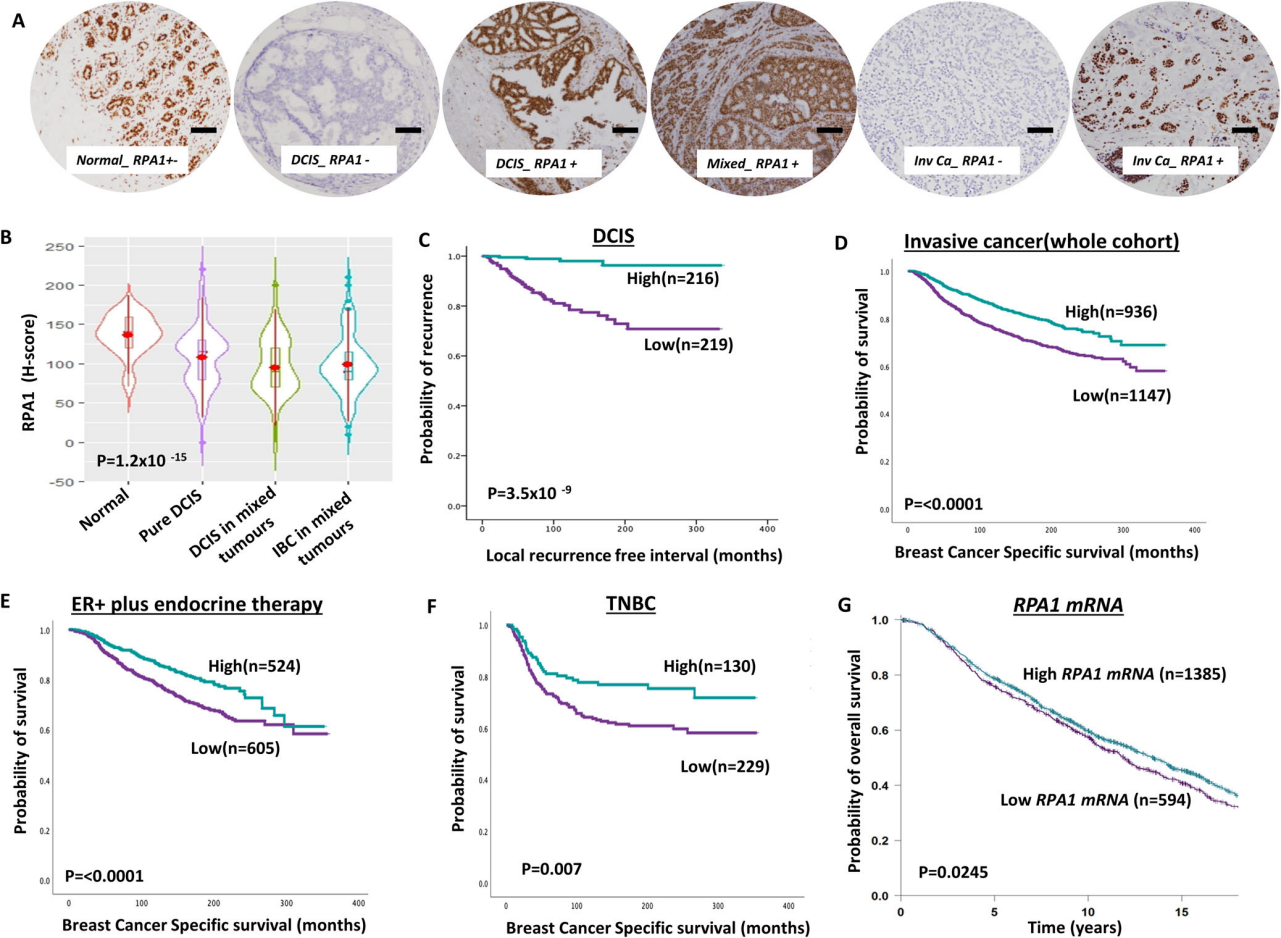
Clinicopathological studies of RPA1 expression in breast cancers. **A** Photomicrographs showing immunohistochemical staining of RPA1 in breast cancers (scale bar “−” = 100 µM). **B** Violin plot shows the mean of RPA1 expression in normal, pure DCIS and DCIS mixed tumours [The red dot represents the median, open red bar in the center represents the interquartile range, the thin red line represents the rest of the distribution, except for points that are “outliers”. On each side of the red line is a kernel density estimation to show the distribution shape of the data. Wider sections of the violin plot represent a higher probability that members of the population will take on the given value; the skinnier sections represent a lower probability.]. **C** Kaplan–Meier curve for RPA1 nuclear protein expression and recurrence-free interval (LRFI) in DCIS (**D**) a Kaplan–Meier curve for RPA1 nuclear protein expression and breast cancer-specific survival (BCSS) in the whole cohort. **E** Kaplan–Meier curve for RPA1 nuclear protein expression and BCSS in ER + cohort with endocrine therapy. **F** Kaplan–Meier curve for RPA1 nuclear protein expression and BCSS in triple negative (TN) in the whole cohort. **G** Kaplan–-Meier curve for RPA1 mRNA expression and breast cancer-specific survival (BCSS) in the whole cohort. Survival rates were determined using Kaplan–Meier method and compared by the log-rank test. All analyses were conducted using Statistical Package for the Social Sciences (SPSS, version 22, Chicago, IL, USA) software for windows. *P* value of less than 0.05 was identified as statistically significant.

RPA1 is the largest subunit of the RPA complex^1–8^. Immunohistochemically, we observed that RPA1 protein was expressed only in the nucleus in both normal and breast cancer tissue. Whereas normal breast tissues have high levels of RPA1 protein expression, the RPA1 level was significantly reduced in DCIS and invasive breast cancers (*P* < 0.0001, Fig. 1A, B). The data suggested that RPA1 downregulation may be an early event during breast cancer pathogenesis. Low RPA1 was observed in 51% (219/434) of DCIS. High nuclear grade DCIS (*P* = 0.01), ER negativity (*P* = 0.006) and PR negativity (*P* = 0.001) were more common in patients with RPA1 deficient DCIS (Supplementary Table 4). The local recurrence-free interval was also significantly reduced in patients with low RPA1 DCIS compared high RPA1 DCIS (*P* < 0.0001) (Fig. 1C).

In IBC, low RPA1 was observed in 55% (1147/2083) of tumours. Low RPA1 was strongly associated with features characteristic of aggressive behaviour including larger size, high grade, cellular de-differentiation and pleomorphism, high mitotic index, high Ki67 index lympho-vascular invasion, lymph node positivity, high-risk Nottingham Prognostic Index (NPI), and with ER negative, PR negative, and triple negative (TNBC) and luminal B breast cancer subtypes (all *P* values <0.001) (Supplementary Table 5).

In the whole cohort, low RPA1 was associated with poor outcome in terms of shorter breast cancer-specific survival (BCSS) (*P* < 0.0001) (Fig. 1D) and distant metastasis-free survival (DMFS) (*P* < 0.0001) (Supplementary Fig. 1A). In Luminal A breast cancers, low RPA1 was associated with shorter BCSS (*P* = 0.006) (Supplementary Fig. 1B) and DMFS (*P* = 0.021) (Supplementary Fig. 1C). In Luminal B tumours, low RPA1 was associated with shorter BCSS (*P* = 0.019) (Supplementary Fig. 1D) and DMFS (*P* = 0.029) (Supplementary Fig. 1E).

In ER + breast cancer patients who received endocrine therapy, low RPA1 was associated with shorter BCSS (*P* < 0.0001) (Fig. 1E) and DMFS (*P* < 0.0001) (Supplementary Fig. 1F) suggesting that low RPA expression may predict limited response to endocrine therapy. In TNBCs, low RPA1 was also associated with shorter BCSS (*P* = 0.007) (Fig. 1F) and DMFS (*P* = 0.009) (Supplementary Fig. 1G). In Her-2 positive breast cancers, RPA expression did not influence survival (Supplementary Fig. 2A, B).

At the transcriptomic level, in the METABRIC cohort, low *RPA1 mRNA* was linked with, Luminal A and Lumina B and Her-2 + PAM50 subtypes compared to basal-like or normal breast-like phenotype (Supplementary Fig. 2C). Low *RPA1 mRNA* was significantly associated with shorter overall survival in the whole cohort (*P* = 0.024) (Fig. 1G), in ER + tumours (*P* = 0.01) (Supplementary Fig. 2D) but not in ER- tumours (*P* = 0.47) (Supplementary Fig. 2E).

#### RPA2

**Fig. 2:**
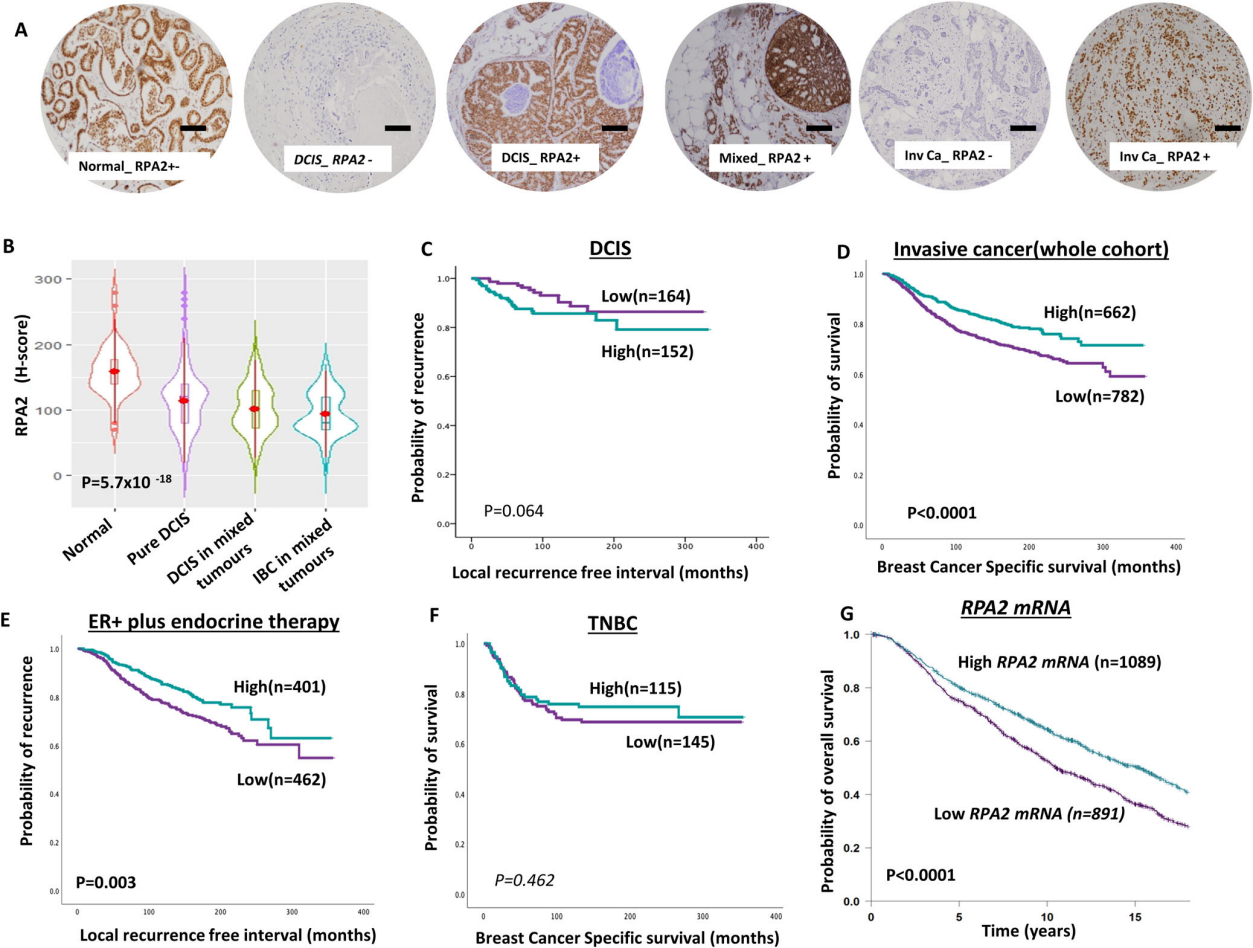
Clinicopathological studies of RPA2 expression in breast cancers. **A** Photomicrographs showing immunohistochemical staining of RPA2 in breast cancers (scale bar “−” = 100 µM). **B** Violin plot shows the mean of RPA2 expression in normal, pure DCIS and DCIS mixed tumours [The red dot represents the median, open red bar in the center represents the interquartile range, the thin red line represents the rest of the distribution, except for points that are “outliers”. On each side of the red line is a kernel density estimation to show the distribution shape of the data. Wider sections of the violin plot represent a higher probability that members of the population will take on the given value; the skinnier sections represent a lower probability.]. **C** Kaplan–Meier curve for RPA2 nuclear protein expression and recurrence-free interval (LRFI) in DCIS (**D**) a Kaplan–Meier curve for RPA2 nuclear protein expression and breast cancer-specific survival (BCSS) in the whole cohort. **E** Kaplan–Meier curve for RPA2 nuclear protein expression and BCSS in ER + cohort with endocrine therapy. **F** Kaplan–Meier curve for RPA2 nuclear protein expression and BCSS in triple negative (TN) in the whole cohort. **G** Kaplan–Meier curve for RPA2 mRNA expression and breast cancer-specific survival (BCSS) in the whole cohort. Survival rates were determined using Kaplan–Meier method and compared by the log-rank test. All analyses were conducted using Statistical Package for the Social Sciences (SPSS, version 22, Chicago, IL, USA) software for windows. *P* value of less than 0.05 was identified as statistically significant.

In normal and breast cancer tissues, RPA2 expression was limited to the nuclei. DCIS and invasive breast cancers showed significantly lower levels of RPA2 compared to normal breast tissue (*P* < 0.0001, Fig. 2A, B). Low RPA2 was observed in 51% (153/302) of DCIS and it was associated with high nuclear grade (*P* = 0.007), comedo necrosis (*P* = 0.009), ER negativity (*P* = 0.001), PR negativity (*P* = 0.003) and triple-negative phenotype (*P* = 0.007) (Supplementary Table 6). RPA2 expression did not influence local recurrence-free interval in DCIS (*P* = 0.064) (Fig. 2C).

**Fig. 3:**
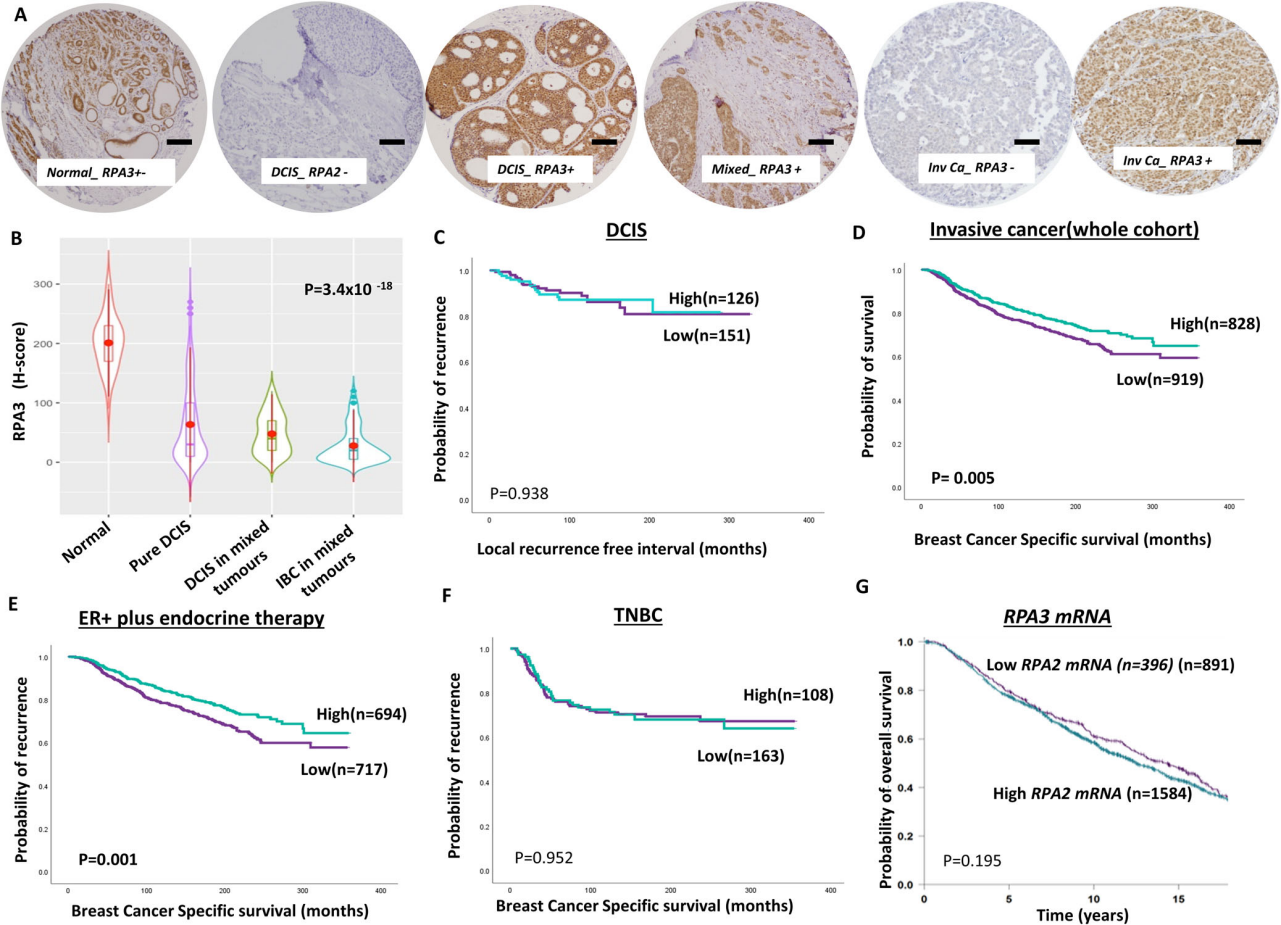
Clinicopathological studies of RPA3 expression in breast cancers. **A** Photomicrographs showing immunohistochemical staining of RPA3 in breast cancers (scale bar “−“ = 100 µM). **B** Violin plot shows the mean of RPA3 expression in normal, pure DCIS and DCIS mixed tumours [The red dot represents the median, open red bar in the center represents the interquartile range, the thin red line represents the rest of the distribution, except for points that are “outliers”. On each side of the red line is a kernel density estimation to show the distribution shape of the data. Wider sections of the violin plot represent a higher probability that members of the population will take on the given value; the skinnier sections represent a lower probability.]. **C** Kaplan–Meier curve for RPA3 nuclear protein expression and recurrence-free interval (LRFI) in DCIS (**D**) a Kaplan–Meier curve for RPA3 nuclear protein expression and breast cancer-specific survival (BCSS) in the whole cohort. **E** Kaplan–Meier curve for RPA3 nuclear protein expression and BCSS in ER + cohort with endocrine therapy. **F** Kaplan–Meier curve for RPA3 nuclear protein expression and BCSS in triple negative (TN) in the whole cohort. **G** Kaplan–Meier curve for RPA3 mRNA expression and breast cancer-specific survival (BCSS) in the whole cohort. Survival rates were determined using Kaplan–Meier method and compared by the log-rank test. All analyses were conducted using Statistical Package for the Social Sciences (SPSS, version 22, Chicago, IL, USA) software for windows. *P* value of less than 0.05 was identified as statistically significant.

In IBC, low RPA2 protein expression was observed in 54% (782/1444) of tumours. Low RPA2 was significantly associated with larger tumours, high grade, de-differentiation, pleomorphism, high mitotic index, high Ki67 index, lympho-vascular invasion, lymph node positivity and high-risk Nottingham Prognostic Index (NPI) (all *P* values ≤0.01) (Supplementary Table 7). In the whole cohort, low RPA2 was associated with poor breast cancer-specific survival (BCSS) (*P* < 0.0001) (Fig. 2D) and distant metastasis-free survival (DMFS) (*P* = 0.001) (Supplementary Fig. 3A). In luminal A, ER + breast cancers, low RPA1 was associated with poor BCSS (*P* = 0.007) (Supplementary Fig. 3B) and DMFS (*P* = 0.007) (Supplementary Fig. 3C). In luminal B ER + breast cancers, there was no significant associations with BCSS or DMFS (Supplementary Fig. 1D, E) and DMFS (*P* = 0.029) (Supplementary Fig. 1E). In ER + breast cancers that received endocrine therapy, low RPA2 was associated with poor BCSS (*P* = 0.003) (Fig. 3E) implying that RPA2 could predict response to endocrine therapy. In TNBCs, low RPA2 was not significantly associated with BCSS (Fig. 3F) and DMFS (Supplementary Fig. 3F). In Her-2 positive breast cancer RPA expression did not influence survival (Supplementary Fig. 4A, B).

At the transcriptomic level, in the METABRIC cohort, low *RPA2 mRNA* was linked with Her-2 + , Luminal A and Lumina B PAM50 subtypes compared to basal-like or normal breast-like phenotype (Supplementary Fig. 4C). In the whole cohort, Low *RPA2 mRNA* was significantly associated with poor overall survival in the whole cohort (*P* < 0.0001) (Fig. 2G), in ER + tumours (*P* < 0.0001) (Supplementary Fig. 4D) and ER- tumours (*P* = 0.013) (Supplementary Fig. 4E).

#### RPA3

RPA3 was expressed in the nucleus and in the cytoplasm. Whereas normal breast tissues have high levels of RPA1 expression, the nuclear RPA3 level was significantly reduced in DCIS and invasive breast cancers (*P* < 0.0001, Fig. 3A, B). Low nuclear RPA3 protein expression was observed in 54% (151/279) of DCIS. Larger size, high nuclear grade, comedo necrosis, ER negativity, PR negativity and HER-2 positive DCIS were more common in patients with RPA3 deficient DCIS (Supplementary Table 8). Low cytoplasmic RPA3 protein expression was observed in 50% (139/279) of DCIS. Larger size, high nuclear grade, comedo necrosis, ER negativity, PR negativity, HER-2 positive and luminal B DCIS were more common in patients with RPA3 deficient DCIS (Supplementary Table 8). RPA3 did not influence local recurrence-free interval (Fig. 3C and Supplementary Fig. 5A).

In invasive breast cancers, low nuclear RPA3 protein expression was observed in 53% (919/1747) of tumours. Low nuclear RPA3 was strongly associated with larger tumours, high grade, high stage, de-differentiation, pleomorphism, high mitotic index, high Ki67 index aggressive breast cancer histological type, lympho-vascular invasion, high-risk Nottingham Prognostic Index (NPI), ER negative, PR negative, triple-negative breast cancers (TNBC) and luminal B breast cancers (all *P* values <0.001) (Supplementary Table 9). In the whole cohort, low nuclear RPA3 protein was associated with poor breast cancer-specific survival (BCSS) (*P* = 0.005) (Fig. 3D). In ER + breast cancers that received endocrine therapy, low nuclear RPA3 protein was associated with poor BCSS (*P* < 0.0001) (Fig. 3E). Low nuclear RPA3 protein did not influence survival in TNBC or Her-2 positive breast cancers (Fig. 3F Supplementary Figs. 5 and 6).

Low cytoplasmic RPA3 protein expression was observed in 55% (965/1747) of tumours. Low cytoplasmic RPA3 protein was strongly associated with larger tumours, high grade, high stage, de-differentiation, high-risk Nottingham Prognostic Index (NPI), ER negative, PR negative, and luminal B breast cancers (all *P* values <0.001) (Supplementary Table 9). In the whole cohort, low cytoplasmic RPA3 protein was associated with poor breast cancer-specific survival (BCSS) (*P* = 0.0004) (Supplementary Fig. 7A) and DMFS (Supplementary Fig. 7B). Low cytoplasmic RPA3 protein did not influence BCSS in TNBC or Her-2 positive breast cancers (Supplementary Figs. 7 and 8).

At the transcriptomic level, in the METABRIC cohort, low *RPA3 mRNA* was associated with normal breast-like phenotype (Supplementary Fig. 8C) but did not influence survival (Fig. 3G and Supplementary Fig. 8D, E).

### RPA and correlation to other DNA repair markers

**Fig. 4:**
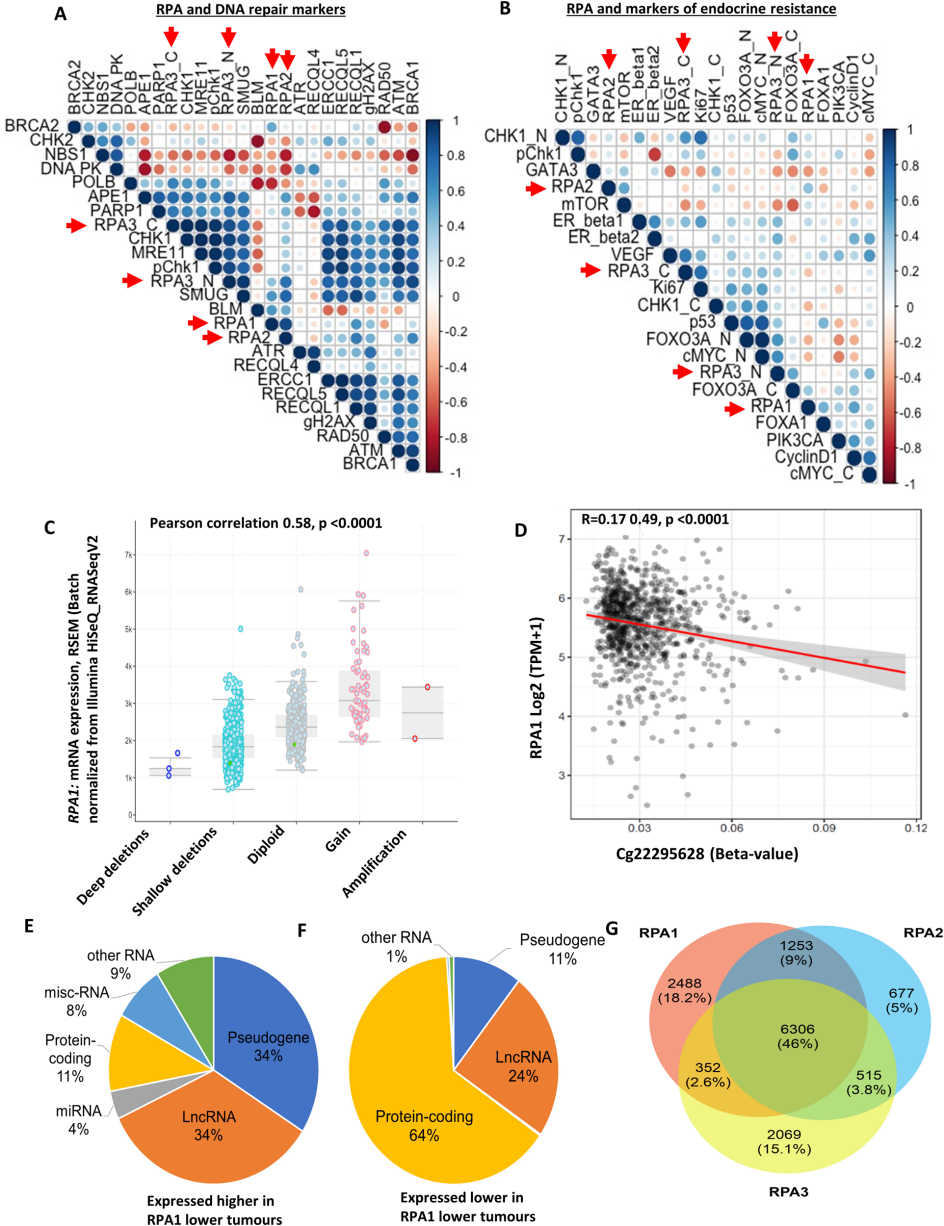
RPA bioinformatics in breast cancer. **A** Correlation matrix showing the correlation between levels of RPA1, RPA2 and RPA3 protein expressions and other DNA repair biomarkers. **B** Correlation matrix showing the correlation between levels of RPA1, RPA2 and RPA3 protein expressions and other endocrine-resistant biomarkers. **C** Comparison of RPA1 gene expression to copy number variation in TCGA-BRCA Pan cancer cohort (*n* = 994). GISTIC analysis is shown for changes in *RPA1* mRNA levels in tumours with copy number variations for TCGA-BRCA Pan cancer cohort (*n* = 994). The expression data was from normalized illumina HiSeq RNA-Seq data. The copy number variations are deep deletions (>2 copies deleted), shallow deletion (few copies altered), diploid, gains (few copies gained), amplification (>2 copies gained). **D** DNA methylation correlations with RPA1 gene expression were performed using SMART App. The beta-values (Illumina HumanMethylation450K) and expression data were from UCSC Xena tools. The CpG correlations shown are for CpG within CpG island in promoter for *RPA1* (see methods sections for more details**)**. The percentage of RNA gene types (Ensembl MART) are shown for non-coding RNAs (lncRNA, pseudogenes, miRNAs and other RNA which include snoRNA, tRNA and MT-RNA) plus protein-coding genes are shown for (**E**) RNAs expressed higher in low *RPA1* tumours (*n* = 10284 confirmed gene types) and **F** RNAs expressed lower in low *RPA1* tumours (*n* = 565 confirmed gene types). **G** Comparison of the differential changes that showed higher expression in low RPA1, RPA2 and RPA3. The RPA components had 46% similarity of the differential changes, with the majority of RPA2 changes like RPA1.

The RPA heterotrimeric complex interacts with several proteins to facilitate DNA repair^1–8^. We have previously immune-profiled several DNA repair markers in the invasive breast cancer cohort^21–33^. We correlated RPA1, 2 and 3 expressions with a panel of DNA repair markers (Fig. 4A). Firstly, there was a positive correlation between RPA1, RPA2 and RPA3. There was a strong positive correlation between RPA1 or RPA2 or RPA3 expression and the expression of proteins involved in DNA damage signalling response (MRE11, RAD50, NBS1, ATM), DNA double-strand break repair (BRCA1, DNA-PKcs), RECQ helicases (BLM, RECQL1, RECQL4, RECQL5), base excision repair (PARP, POLB, SMUG1), nucleotide excision repair (ERCC1) and cell cycle regulation (CHK1, CHK2, pCHK1, pCHK2) (all *P* values <0.001) (Supplementary Tables 10–12) (Fig. 4A).

### RPA and correlation with markers of endocrine resistance

In ER + breast cancers that received endocrine therapy, low RPA1, 2 or 3 was associated with poor BCSS and DMSF. We therefore explored whether RPA1, 2 or 3 expression correlates with known markers of endocrine resistance. As shown in Fig. 4B, there was positive correlation between RPA complex and markers of endocrine resistance including mTOR, FOXO3A, PIK3CA, CyclinD1, Ki67 and ERβ1 (Fig. 4B) (Supplementary Tables 13–15).

### Genomic and transcriptomics analysis of RPA in breast cancers

Utilising cBioportal to examine the TCGA-BRCA cohort, we observed that mutations in RPA1, RPA2 and RPA3 were very rare (3 mutations only in 1055 patients). GISTIC analysis is shown in Fig. 4C and Supplementary Fig. 9A, B for changes in mRNA levels in tumours with *RPA* gene copy number variations for TCGA-BRCA Pan cancer cohort (*n* = 994). There was a significant positive correlation between copy number changes and gene expression for *RPA1* (Pearson correlation 0.58, *P* < 0.0001) (Fig. 4C)*, RPA2* (0.62, *P* < 0.0001) (Supplementary Fig. 9A) and *RPA3* (0.49, *P* < 0.0001) (Supplementary Fig. 9B) genes. The CpG correlations shown are for CpG within CpG island in promoter for RPA1 (Fig. 4D), RPA2 (Supplementary Fig. 10A) and RPA3 (Supplementary Fig. 10B) genes. At the promoter level, there was a strong positive association between *RPA1* promoter methylation and *RPA1* gene expression (*P* < 0.001, Fig. 4D) but not for *RPA2* or *RPA3* (Supplementary Fig. 10A, B). At the intragenic level, we observed a significant association for *RPA2* (*P* < 0.001, Supplementary Fig. 10D) but not for *RPA1* or *RPA3* (Supplementary Fig. 10C, E). We then evaluated the expression patterns of non-coding RNAs (lncRNA, pseudogenes and novel transcripts, miRNAs) and coding genes in RPA low or high expressing tumours. RNAs with high expression in low *RPA1* tumours (*n* = 10797) is shown in Fig. 4E and genes with expression in high *RPA1* tumours (*n* = 571) is shown in Fig. 4F. Interestingly, pseudogene expression (34%) and lncRNA/novel transcripts expression (34%) was higher in RPA1 low tumours compared to *RPA1* high tumours (11% and 24%, respectively) (Figs. 4E, F). These findings were also similar for *RPA2* and *RPA3* (Supplementary Fig. 11A–D) suggesting that low RPA complex leads to increase in non-coding RNA expression. This observation is likely related to the role of RPA in retro-transposition. Comparison of the differential changes that showed higher expression in low RPA1, RPA2 and RPA3 is shown in Fig. 4G. The RPA components had 46% similarity of the differential changes, with the majority of RPA2 changes like RPA1. We then proceeded to investigate expression patterns of functional genes in tumours. Differential genes associated with low RPA complex expressing tumours (Q1, *n* = 273) and high RPA complex expressing tumours (Q4, *n* = 273) were obtained utilising TCGA-BRCA data (*n* = 1090). The data was sorted by genes that show log2 fold change ≥1 and FDR corrected *P* < 0.05 (summarized in supplementary data 1). Pathway analysis of genes that were expressed higher with low RPA1, low RPA2 and low RPA3 is shown in Supplementary data 2. Pathway analysis of the RPA complex identified genes that were expressed higher with low RPA quartile (Q1) (all FDR < 0.05) and included those involved in steroid hormone biosynthesis, chemical carcinogenesis and drug metabolism (Supplementary Data 2, Supplementary Fig. 11E and Supplementary Table 16). The genes that were coming up in many of the pathways was the UDP glucuronosyltransferase (UGT) family, specifically the UGT1A family members, UGT1A3, UGT1A6-9 (FDR < 0.05, log2 fold change of 1.16, 1.04, 3.00, 2.30 and 1.2, respectively). UGT are involved in the glucuronidation of hormones and drugs, which reduces the biological function of the molecules and leads to elimination from the cell^34^. In cancer, UGTs have altered expression, are linked to drug resistance and drug cytotoxicity^35–37^.

Taken together, the data provide clinical evidence that RPA deficiency promote an aggressive breast cancer phenotype and adversely impact survival outcomes. To evaluate whether RPA deficient breast cancers can be targeted by precision oncology, we conducted pre-clinical studies in a panel of breast cancer cell lines.

### Pre-clinical studies

We first expression profiled MCF10A (normal), MCF10A-DCIS, MCF-7 (ER + ), and MDA-MB-231 (triple negative) for RPA1, 2 and 3 expressions. Robust expression of RPA1 (Supplementary Fig. 12A, B) and RPA2 (Supplementary Fig. 12C, D) was observed in MCF-7, MDA-MB-231 but RPA3 expression was low in all cell lines (Supplementary Fig. 12E, F). We proceeded to deplete RPA1 and RPA2 by siRNAs in MCF7 and MDA-MB-231 cells and investigate cisplatin or Olaparib sensitivity.

#### RPA1 depleted breast cancer cells are sensitive to cisplatin chemotherapy

**Fig. 5:**
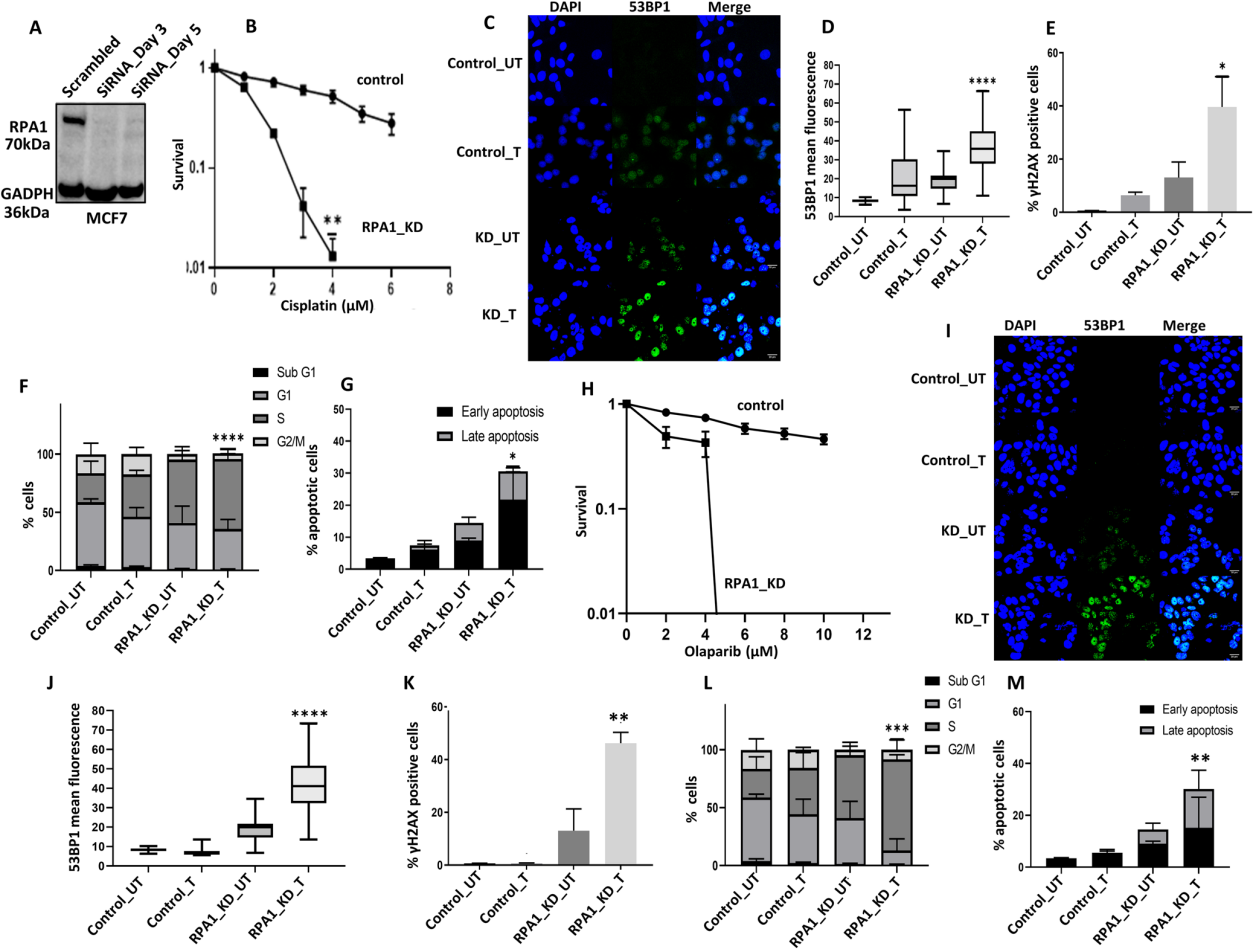
RPA1 depletion and cisplatin or Olaparib sensitivity in breast cancer cells. **A** RPA1 siRNA knock down in MCF7 cells. Lysates were collected at day3 and day5. **B** Clonogenic survival assay for cisplatin sensitivity in MCF7 cells control and MCF7_RPA1_KD cells. **C** Representative photo micrographic images for immunofluorescence staining of 53BP1 in MCF7 control MCF7_RPA1_KD cells treated with Cisplatin (5 μM) for 24 h. **D** Quantification of 53BP1 nuclear fluorescence by ImageJ software. **E** Quantification of γH2AX positive cells by flow cytometry. **F** Cell cycle analysis by flow cytometry. **G** Annexin V analysis for apoptotic cells in MCF7 control and RPA1_knock down cells treated with 5 μM cisplatin for 24 h. **H** Clonogenic survival assay for Olaparib sensitivity in MCF7 cells control and MCF7_RPA1_KD cells. **I** Representative photo micrographic images for immunofluorescence staining of 53BP1 in MCF7 control MCF7_RPA1_KD cells treated with Olaparib (6 μM) for 24 h. **J** Quantification of 53BP1 nuclear fluorescence by ImageJ software. **K** Quantification of γH2AX positive cells by flow cytometry. **L** Cell cycle analysis by flow cytometry. **M** Annexin V analysis for apoptotic cells in MCF7 control and RPA1_knock down cells treated with 6 μM Olaparib for 24 h. Figures are representative of 3 or more experiments. Statistical analysis was conducted as on GraphPad Prism7 software. To compare between two groups, Student *T* tests analysis was performed. One-way ANOVA was performed to compare between more than two groups (variances analyses). Two-way ANOVA was used to analyse two variables such as Annexin V analysis and cell cycle analysis. All experiments were expressed as means ± standard deviation S.D. of three independent experiments. Error bars represent standard error of mean between experiments. UN untreated, T treated. *P value <0.05, **P value <0.001, ***P value <0.0001.

We generated transient knockdowns (KD) of RPA1 using siRNAs in MCF-7 cells (Fig. 5A). In clonogenic assays, RPA1_KD_MCF7 cells (Fig. 5B) were significantly sensitive to platinum compared to scrambled control. Increased cytotoxicity in RPA1_KD_MCF7 cells was associated with increased 53BP1 foci accumulation (a marker of DSB accumulation) (Fig. 5C, D) compared to control cells. Similarly, γH2AX immunofluorescence was increased in RPA1_KD_MCF7 cells compared to controls (Fig. 5E and Supplementary Fig. 12G) confirming DSB accumulation. Increased DSB was associated S-phase cell cycle arrest (Fig. 5F and Supplementary Fig. 12H) and increased apoptosis (Fig. 5G and Supplementary Fig. 12I) compared to scrambled controls. For further validation, we also depleted RPA1 in MDA-MB-231 cells (Supplementary Fig. 13A). As shown in Supplementary Fig. 13B, RPA1_KD_ MDA-MB-231 cells showed increased platinum sensitivity compared to scrambled controls. Increased sensitivity to cisplatin in RPA1_KD_ MDA-MB-231 cells was also associated with DSB accumulation (Supplementary Fig. 13C), G1-phase arrest and increased sub-G1 population (Supplementary Fig. 13D) and increased apoptotic cells (Supplementary Fig. 13E). We additionally validated cisplatin sensitivity using another siRNA construct for RPA1 depletion. As shown in Supplementary Fig. 14A–D, RPA1 depletion increased platinum sensitivity in MCF-7 and MDA-MB-231 cells.

#### Olaparib induced synthetic lethality in RPA1 deficient breast cancer cells

RPA deficient cells will accumulate single-strand DNA breaks (SSB) which will activate PARP, a key protein for the coordination of SSB repair. PARP blockade by Olaparib will not only inhibit PARP biochemical activity but will also trap PARP at replication forks leading onto accumulation of DSBs, repair of which is impaired in RPA deficient cells. DSB accumulation eventually leads to apoptotic cell death. We tested this hypothesis in RPA1 depleted MCF7 and MDA-MB-231 cells.

RPA1_KD_MCF7 cells were extremely sensitive to Olaparib therapy compared to scrambled controls (Fig. 5H). Significantly increased 53BP1 (Fig. 5I, J) and γH2AX immunofluorescence (Fig. 5K) in RPA1_KD_MCF7 cells compared to controls confirmed DSB accumulation. Increased DSB was associated S-phase cell cycle arrest (Fig. 5L) and increased apoptosis (Fig. 5M) compared to scrambled controls. In RPA1_KD_ MDA-MB-231 cells, similarly, extreme sensitivity to Olaparib was evident (Supplementary Fig. 13F) which was associated with DSB accumulation (Supplementary Fig. 13G), G1 arrest (Supplementary Fig. 13H) and increased apoptosis (Supplementary Fig. 13I).

The data presented so far suggests that RPA1 deficient cells could be suitable for PARP blockade directed synthetic lethality strategy. We the tested whether a similar approach would be feasible in RPA2 deficient breast cancer cells.

#### Cisplatin or Olaparib is selectively toxic in RPA2 deficient breast cancer cells

**Fig. 6:**
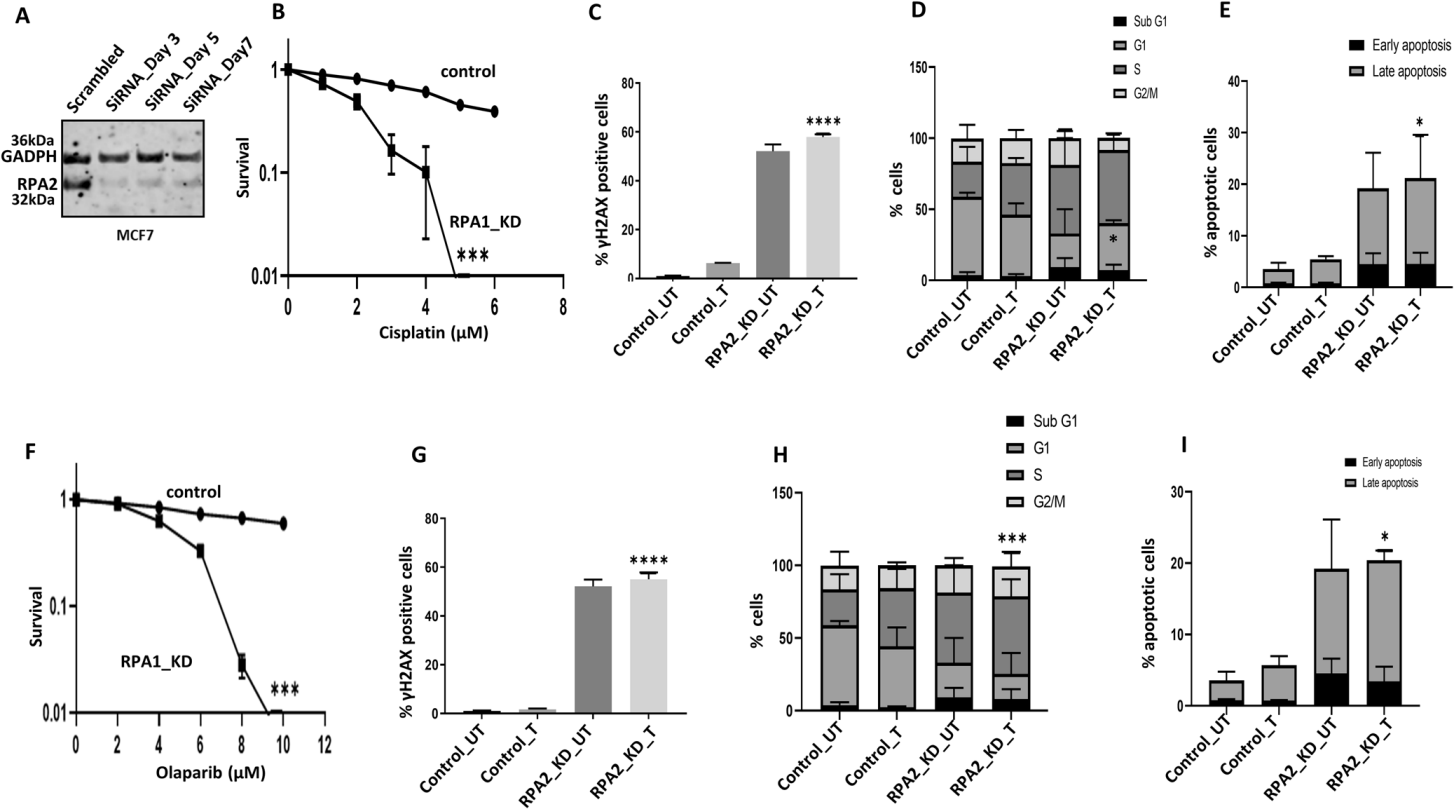
RPA2 depletion and cisplatin or Olaparib sensitivity in breast cancer cells. **A** RPA2 siRNA knock down in MCF7 cells. Lysates were collected at day3 and day5. **B** Clonogenic survival assay for cisplatin sensitivity in MCF7 cells control and MCF7_RPA2_KD cells. **C** Quantification of γH2AX positive cells by flow cytometry. **D** Cell cycle analysis by flow cytometry. **E** Annexin V analysis for apoptotic cells in MCF7 control and RPA2_knock down cells treated with 5 μM cisplatin for 24 h. **F** Clonogenic survival assay for Olaparib sensitivity in MCF7 cells control and MCF7_RPA2_KD cells. **G** Quantification of γH2AX positive cells by flow cytometry. **H** Cell cycle analysis by flow cytometry. **I** Annexin V analysis for apoptotic cells in MCF7 control and RPA2_knock down cells treated with 6 μM Olaparib for 24 h. Statistical analysis was conducted as on GraphPad Prism7 software. To compare between two groups, student- T-tests analysis was performed. One-way ANOVA was performed to compare between more than two groups (variances analyses). Two-way ANOVA was used to analyse two variables such as Annexin V analysis and cell cycle analysis. All experiments were expressed as means ± standard deviation S.D. of three independent experiments. Error bars represent standard error of mean between experiments. UN untreated, T treated. *P value <0.05, **P value <0.001, ***P value <0.0001.

We generated transient knockdowns (KD) of RPA2 using siRNAs in MCF-7 cells (Fig. 6A). RPA2_KD_MCF7 cells were sensitive to cisplatin compared to scrambled control (Fig. 6B). Increased cytotoxicity in RPA2_KD_MCF7 cells was associated with DSB accumulation (Fig. 6C), S-phase cell cycle arrest (Fig. 6D) and increased apoptosis (Fig. 6E) compared to scrambled controls. We also depleted RPA2 in MDA-MB-231 cells (Supplementary Fig. 15A). As shown in Supplementary Fig. 15B, RPA2_KD_ MDA-MB-231 cells were sensitive to cisplatin therapy and was associated with DSB accumulation (Supplementary Fig. 15C), S1-phase arrest (Supplementary Fig. 15D) and increased apoptotic cells (Supplementary Fig. 15E). We also validated cisplatin sensitivity using another siRNA construct for RPA2 depletion (Supplementary Fig. 16A–D).

RPA2_KD_MCF7 cells were extremely sensitive to Olaparib therapy compared to scrambled controls (Fig. 6F) and associated with DSB accumulation (Fig. 6G), S-phase arrest (Fig. 6H) and apoptosis (Fig. 6I). In RPA2_KD_ MDA-MB-231 cells, similarly, were sensitivity to Olaparib (Supplementary Fig. 115F) which was associated with DSB accumulation (Supplementary Fig. 15G), S-phase arrest (Supplementary Fig. 15H) and apoptosis (Supplementary Fig. 15I).

## Discussion

DCIS^38^ accounts for up to 20% of all breast cancers^39^. Over 50% of patients with high-grade DCIS, if untreated, may progress to invasive carcinoma in less than 5 years. On the other hand, for low-grade DCIS, the risk of developing invasive disease is lower (35–50% over 40 years)^39^. Whilst surgery (mastectomy or wide local excision), with or without adjuvant radiotherapy aim to prevent DCIS from progressing to invasive cancer, there is currently no personalized approach to tailor treatments. The development of markers to define aggressive DCIS sub-types is an area of unmet need. Genomic instability and the consequent somatic mutation accumulation may influence DCIS biology^40^. Furthermore, persistent, and impaired genomic stability in invasive cancers can facilitate aggressive clinical behaviour. In the current study we show, for the first time, that RPA protein loss is frequent in breast cancer including its precursor lesion (DCIS) and is linked to aggressive phenotypes. Low RPA was associated with clinicopathological features characteristic of aggressive behaviour and with poor outcome. At mRNA level, low *RPA* expression was also linked with poor survival. The association between low RPA and poor outcome was maintained in patients who received endocrine therapy suggesting that these tumours have limited response to endocrine therapy. This observation was supported at the molecular level as low RPA tumours showed overexpression of genes involved in steroid hormone biosynthesis, in addition to genes involved in chemical carcinogenesis, and drug metabolism a feature that could contribute to drug resistance.

Loss of expression of RPA promote genomic instability given its critical role during replication, checkpoint regulation, DNA repair, telomere maintenance and retro-transposition. Accordingly, at the protein level, we observed a strong positive correlation between RPA expression and the expression of proteins involved in genomic stability including DNA damage signalling response (MRE11, RAD50, NBS1, ATM), DNA double-strand break repair (BRCA1, DNA-PKcs), RECQ helicases (BLM, RECQL1, RECQL4, RECQL5), base excision repair (PARP, POLB, SMUG1), nucleotide excision repair (ERCC1) and cell cycle regulation (CHK1, CHK2, pCHK1, pCHK2).

Given the multifunctional role of RPA, we speculated that its downregulation may have genome wide impact that could influence cancer progression and prognosis. Therefore, to understand the mechanisms of low RPA expression and the transcriptomic consequence of such downregulation, we conducted detailed bioinformatics studies. At the transcriptomic level, low RPA tumours had overexpression of pseudogenes and lncRNA, a feature that has been associated with genomic instability^41,42^ and likely related also to the role of RPA in retro-transposition^9^. Taken together, our data suggest that RPA loss in DCIS and invasive cancers could promote genomic instability leading onto aggressive phenotypes and poor survival. Dysregulation of RPA expression has been reported in other solid tumours. In a study of 130 clinical colorectal cancers^15^, RPA1 and RPA2 overexpression was shown to have poor prognostic significance in patients. In another small study of 66 astrocytomas^16^, RPA2 overexpression was associated with poor survival in grade IV tumours. In 48 oesophageal cancer samples^12^, RPA1 and RPA2 was elevated in late stage disease. RPA1 Overexpression was associated with poor survival outcomes in that study^12^. On the other hand, in 156 bladder cancers^17^, low RPA1 and low RPA2 was associated with poor survival in patients. Interestingly in a study in breast cancer patient^43^, autoantibodies to RPA2 were detected before diagnosis of invasive tumours and anti-RPA2 antibodies were also prevalent in DCIS indicating a role for RPA during breast cancer pathogenesis^43^.

Platinum sensitivity is a feature of breast cancer cells that harbour BRCA germ-line deficiency or those that manifest homologous recombination deficiency (HRD)^44^. RPA has key roles in DNA replication, repair, recombination, NER and Fanconi anaemia pathway. Accordingly, here we have shown that RPA deficient breast cancer cells are sensitive to cisplatin therapy. More importantly, we have demonstrated that RPA deficient cells can also be targeted for synthetic lethality using PARP inhibitor Olaparib. We speculate model for synthetic lethality as follows: (a) RPA deficient cells will accumulate single-strand DNA breaks (SSB); (b) SSB will activate PARP, a key protein for the coordination of SSB repair; (c) PARP blockade by Olaparib will not only inhibit PARP biochemical activity but will also trap PARP at replication forks leading onto accumulation of DSB; (d) in RPA deficient cells, DSB repair is also impaired resulting in DSB accumulation and cell death.

We conclude that RPA, a critical player during DNA replication, recombination and repair is downregulated in a proportion of DCIS and invasive breast cancers. Such RPA deficient tumours can be exploited for precision oncology strategy.

## Methods

### Clinical study

#### RPA protein expression level in breast ductal carcinoma in situ (DCIS)

A total of 776 patients with pure DCIS diagnosed between 1987 and 2012 were identified from the National Health System (NHS) database of the Nottingham University Hospitals. A cohort of 239 DCIS that co-exist with invasive breast cancer (IBC) as well as 50 normal breast tissues were also identified. Patients’ demographics including tumor grade, tumor size, age, menopausal status, screening or symptomatic presentation DCIS, histological type, presence of comedo necrosis, Paget’s disease, associated lobular carcinoma in situ (LCIS) as well as local recurrence and recurrence-free interval (defined as time in months from diagnosis to the development of local recurrence) were collected. All patients received surgery. TMAs were constructed and immune-stained for RPA1, RPA2 and RPA3. Not all cores within the TMA were suitable for IHC analysis due to missing cores or absence of tumour cells.

#### RPA protein expression level in invasive breast cancer

The study was performed in a large series of 4221 invasive breast cancer cases treated at Nottingham University Hospitals (NUH) between 1986 and 2006. All patients were treated in a single institution and have been investigated in a wide range of biomarker studies. Supplementary Table S2 summarizes patient demographics. Patients received standard surgery (mastectomy or wide local excision) with radiotherapy. Prior to 1989, patients did not receive systemic adjuvant treatment (AT). After 1989, AT was scheduled based on prognostic and predictive factor status, including NPI, oestrogen receptor-α (ER-α) status, and menopausal status. Patients with NPI scores of <3.4 (low risk) did not receive AT. In pre-menopausal patients with NPI scores of ≥3.4 (high risk), classical Cyclophosphamide, Methotrexate, and 5-Flourouracil (CMF) chemotherapy was given; patients with ER-α positive tumours were also offered HT. Postmenopausal patients with NPI scores of ≥3.4 and ER-α positivity were offered HT, while ER-α negative patients received classical CMF chemotherapy. Median follow up was 111 months (range 1 to 233 months). Survival data, including breast cancer-specific survival (BCSS) and the development of loco-regional and distant metastases (DM), was maintained on a prospective basis. Breast cancer-specific survival (BCSS) was defined as the number of months from diagnosis to the occurrence of BC-related death. DM-free survival was defined as the number of months from diagnosis to the occurrence of DM relapse. Survival was censored if the patient was still alive at the time of analysis, lost to follow-up, or died from other causes.

This work obtained ethics approval from the Northwest–Greater Manchester Central Research Ethics Committee under the title; Nottingham Health Science Biobank (NHSB), reference number 15/NW/0685. Informed consent was obtained from all individuals prior to surgery to use their tissue materials in research. All samples used in this study were pseudo anonymized and collected prior to 2006 and stored in compliance with the UK Human Tissue Act. The Reporting Recommendations for Tumor Marker Prognostic Studies (REMARK) criteria, recommended by McShane et al.^45^, were followed throughout this study.

#### Tissue microarray (TMA) and immunohistochemistry (IHC)

Tissue microarrays (TMAs) were constructed from both study cohorts (DCID and IBC) where a representative core were taken from each donor FFPE tissue block into recipient TMA blocks. The staining was conducted on 4μm thick sections. Immunohistochemical staining was conducted using the Novolink Max Polymer Detection System (RE7280-K: 1250 tests), and the Leica Bond Primary Antibody Diluent (AR9352), each used according to the manufacturer’s instructions (Leica Microsystems). The tissue slides were deparaffinised with xylene and then rehydrated through five decreasing concentrations of alcohol (100%, 90%, 70%, 50% and 30%) for 2 min each. Pre-treatment antigen retrieval was performed on the TMA sections using sodium citrate buffer (pH 6.0) and heated for 20 min at 95 °C in a microwave (Whirlpool JT359 Jet Chef 1000 W). A set of slides were incubated for 1 h at room temperature with rabbit monoclonal RPA1 (dilution 1:100, Abcam-ab79398), mouse monoclonal RPA2 (1:100, Abcam-ab2175) along with a rabbit polyclonal RPA3 (dilution 1:50, Abcam-ab97436) (Supplementary Table 3).

#### Evaluation of immunohistochemical staining

Whole field inspection of the core was conducted. The subcellular localisation (nucleus, cytoplasm, cell membrane) of each marker was identified. Intensities of subcellular compartments were each assessed and grouped as follows: 0 = no staining, 1 = weak staining, 2 = moderate staining, 3 = strong staining. The percentage of tumour cells in each category was estimated (0–100%). Histochemical score (H-score) (range 0–300) was calculated by multiplying the intensity of staining and the percentage of staining.

As the continuous H scores of the protein expression in each sub-localization were non-normally distributed, median was used to categorise the scores into low, negative/high, positive groups. For consistency and to avoid potential bias, the median for each localisation was used. A median cut-off for H-score was chosen for high or low expression. A median H-score of ≤100 was used as the cut-off for low RPA1 nuclear expression. A median H-score of ≤70 was used as the cut-off for low RPA2 nuclear expression. A median H-score ≤40 was used as the cut-off for low RPA3 nuclear expression. A median H-score ≤40 was used as the cut-off for low RPA3 cytoplasmic expression. No sub-scores were considered in Histochemical-score as the percentage was used as a continuous scale and multiplied in each staining intensity.

#### Statistical analysis

Association with clinical and pathological parameters using categorised data was examined using Chi-squared test. All tests were two-tailed. Survival rates were determined using Kaplan–Meier method and compared by the log-rank test. All analyses were conducted using Statistical Package for the Social Sciences (SPSS, version 22, Chicago, IL, USA) software for windows. *P* value of less than 0.05 was identified as statistically significant.

#### Transcriptomic analyses

Prognostic significance of *RPA1 mRNA* (HGNC ID 10289), *RPA2 mRNA* (HGNC ID 10290) and *RPA3 mRNA* (HGNC ID 10291) was evaluated in a publicly available microarray dataset from 1980 breast cancer patients (cohort 1)^46^.

#### Bioinformatics

cBioportal was utilised to analyse the mutations and for correlation of clinic-pathologic features to the RPA gene expression^47^. We also analysed the DNA methylation status of the RPA genes using The SMART App, which utilises the Infinium 450methylation array^48^. DNA methylation (promoter and intragenic) correlations with RPA gene expression were then performed using SMART App. The beta-values (Illumina HumanMethylation450K) and expression data were from UCSC Xena tools. The TCGA breast cancer (BRCA) RNAseq expression data was obtained from GDC (https://portal.gdc.cancer.gov/). The specimens (*n* = 1090) were firstly ranked from lowest to highest expression for RPA1, RPA2 and RPA3. The sum of the ranks for the three RPA components were then calculated and divided into quartiles, with Q1 (*n* = 273) having the lowest expression of RPA components and Q4 (*n* = 272) having the highest. Differentially expressed genes between Q1 and Q4 were identified using DESeq2^49^. Differential genes were taken forward to pathway analysis if they obtained significant change of log2 fold of 1 and above, FDR-p value <0.05. Pathway analysis was performed using WebGestalt, utilising KEGG database^49^. Significant pathways showed FDR-p value <0.05.

### Pre-clinical study

#### Cell lines and tissue culture

MDA-MB-231, MCF7 and SKBR3 were purchased from American Type Culture Collection (ATCC, Manassas, USA). MDA-MB-231 and MCF7 were grown in RPMI (R8758, Merck, UK). SKBR3 cell line was cultured in McCoy’s 5 A medium (Sigma Life Science, SLCB4463, USA). All the mediums were supplemented with 10% foetal bovine serum (F4135, Merck, UK) and 1% Penicillin–Streptomycin (P4333, Merck, UK). MCF10A and MCF10DCIS cells were cultured in DMEM-F12 supplemented with 10% horse serum, 5 mg/mL insulin, 1 mg/mL cholera toxin, and 100 mg/mL EGFR, 5 mg/mL hydrocortisone and 1% penicillin–streptomycin. Cell lines were routinely tested for mycoplasma contamination every 4 weeks.

#### Compounds and reagents

Cisplatin solution (1 mg/ml) was obtained from the Department of Pharmacy, Nottingham University Hospitals, Nottingham, UK. Olaparib (AZD2281) was purchased from Selleckchem, UK.

#### Western blot analysis

Cells were harvested and lysed in RIPA buffer (R0278, Sigma.UK) with the addition of protease cocktail inhibitor (P8348, Sigma, UK), phosphatase inhibitor cocktail 2 (P5726, Sigma, UK) and phosphatase inhibitor cocktail 3 (P0044, Sigma) and stored at −20 °C. Proteins were quantified using BCA Protein Assay kit (23225, Thermofisher, UK). Samples were run on SDS-bolt gel (4–12%) bis-tris. Membranes were incubated with primary antibodies as follows: RPA1 (1:1000, ab79398), RPA2 (1:1000, ab2175), RPA3 (1:1000, ab97436. Membranes then were washed and incubated with Infrared dye-labelled secondary antibodies (LiCor) [IRDye 800CW Donkey Anti-Rabbit IgG (926-32213) and IRDye 680CW Donkey Anti-Mouse IgG (926-68072)] at dilution of 1:10,000 for 1 h. Membranes were scanned with a LiCor Odyssey machine (700 and 800 nm) to determine protein levels. Uncropped western blot gels are shown in supplementary Figs. 17–19.

#### Transient knockdowns of RPA

RPA1 (ID S12130) and the validation construct RPA1 (S12132) and RPA2 (ID S12127) and the validation construct RPA2 (S12128) siRNAs, RPA3 oligonucleotides were obtained from Invitrogen. Lipofectamine 3000 reagent (L3000015, Invitrogen, UK) was used according to the manufacturer’s protocol. Briefly, cells were seeded at 50–60% confluency in T25 flasks overnight. Cells were transfected with 20 nM of siRNA oligonucleotide or scrambled SiRNA oligonucleotide control (4390843, Thermofiher) in Opti-MEM media (31985-062, Gibco). Transfection efficiency was confirmed using western blot.

#### Clonogenic assays

In the clonogenic assay, 200–400 cells/cm^2^ were seeded in 6-well plates and left at 37 °C in a 5% CO_2_ atmosphere. Cisplatin (Kindly provided by Nottingham University Hospital) or Mirin (M9948, Sigma, UK) were added at the indicated concentrations and the plates were left at 37 °C in a 5% CO_2_ atmosphere for 14 days. Later the plates were washed with PBS, fixed and stained and colonies were counted.

#### Functional studies

In all, 1 × 10^5^ cells per well were seeded in six-well plates and left overnight at 37 °C in a 5% CO_2_ atmosphere. After 24 h, 5 µM of Cisplatin or 18 µM or 25 µM of Mirin were added to cells and incubated for 24 h and 48 h. Cells then were collected by trypsinization, washed with ice cold PBS, and fixed in 70% ethanol for 1 h at −20 °C. After removal of the fixative solution by centrifugation, for DNA double-strand break analysis, cells were stained with 2 mg/ml of phospho-Histone (γH2AX) Ser139 (16202 A, Millipore, UK). For cell cycle analysis, cells were treated with 20 mg/ml RNase A (12091021, Invitrogen) and then 10 mg/ml Propidium Iodide (P4170, Sigma Aldrich) was added to determine the cell cycle distribution. The samples were analysed on a Beckman-Coulter FC500 flow cytometer using a 488 nm laser for excitation and emission data for PI collected using a 620 nm bandpass filter (FL3) and a 525 nm bandpass filter (FL1) for FITC-anti-phospho-Histone H2A.X. For the Apoptosis assay, cells were analysed using Annexin V detection kit (556547, BD Biosciences). Briefly, cells were trypsinized, washed with PBS and the cellular pellet was re-suspended in Annexin Binding Buffer (1x). Then 2.5 ml of FITC Annexin V and 2.5 ml of Propidium Iodide were added to the cells. After incubation 300 ml of Annexin Binding Buffer (1x) was added to each tube. Samples were analysed on a Beckman-Coulter FC500 flow cytometer. Data were analysed by Weasel software. Graphical representation and statistical analysis were performed in GraphPad Prism7 (GraphPad, La Jolla, USA).

#### Immunofluorescence staining

Cells were seeded on the cover slips overnight, then fixed with 4% paraformaldehyde (8187085000, Sigma) for 30 min and whished with PBS. permeabilized with 0.1% triton (HFH10, Thermofisher) for 30 min. Cells were blocked with 3% BSA (A7906, Sigma) for 1 h. Cells were incubated with 53PB1 (1:100) overnight at 4 °C. Slides then washed and incubated with goat anti-Rabbit (A16129, Invitogen, UK) and goat anti-mouse (A11029, Invitogen, UK) for 1 h. Imaging was carried out using Leica confocal microscope. For analysis at least 100 cells per slides were counted.

### Statistical analysis

Statistical analysis was conducted as on GraphPad Prism7 software. To compare between two groups, Student *T* tests analysis was performed. One-way ANOVA was performed to compare between more than two groups (variances analyses). Two-way ANOVA was used to analyse two variables such as Annexin V analysis and cell cycle analysis. All experiments were expressed as means ± standard deviation SD of three independent experiments. *P* values <0.05 = *, *P* value <0.01 = ** & *P* value <0.001 = ***.

## Supplementary information

Supplementary Tables_Figures_Figure legends

Data set 1

Data set 2

## Data Availability

Data supporting the study can be found in the supplementary information file, and the corresponding author can make any materials available upon request. Aggregate data from the referenced datasets are available from the corresponding author on reasonable request. Primary datasets generated during the study are available in Supplementary Data 1 and 2. Referenced datasets analyzed in the study are described in methods and accession codes are as follows; E-MTAB-365, E-TABM-43, GSE11121, GSE12093, GSE12276, GSE1456, GSE16391, GSE16446, GSE16716, GSE17705, GSE17907, GSE18728, GSE19615, GSE20194, GSE20271, GSE2034, GSE20685, GSE20711, GSE21656, GSE22093, GSE25066, GSE2603, GSE26971, GSE29044, GSE2090, GSE31448, GSE32646, GSE3494, GSE36771, GSE37946, GSE41998, GSE43358, GSE43365, GSE45255, GSE4611, GSE46184, GSE46184, GSE48390, GSE50948, GSE5327, GSE58812, GSE61304, GSE65194, GSE6532, GSE69031, GSE7390, GSE76275, GSE78958, GSE9195, GSE 19783 and GSE 40267.
