# Supplementary material for: Unravelling the clinicopathological and functional significance of replication protein A (RPA) heterotrimeric complex in breast cancers
Source: NPJ Breast Cancer. 2023 Mar 30;9:18. doi: 10.1038/s41523-023-00524-3 (PMC10063624; doi:10.1038/s41523-023-00524-3)

Supplementary Table 1: Patient demographics in pure DCIS cohort (n=453).

Parameter	Groups	No
Age	≤45 years	57
	>45 years	396
Presentation	Screening	217
	symptomatic	236
Extent	Localized	305
	Diffused	67
Size	≤20mm	195
	>20mm	255
Grade	Low	60
	Moderate	117
	High	276
Comedo/ Necrosis	No.	154
	Yes	299
DCIS type	Pure DCIS	453
	DCIS + invasive	196
LCIS	No	411
	Yes	42
Paget's.	No	276
	Yes	21
ER status	Negative	109
	Positive	308
PR status	Negative	177
	Positive	246
Treatment	Mastectomy	260
	Breast conserving surgery +RT	193
	Breast conserving surgery	123

Supplementary Table 2. Patient demographics in invasive breast cancer cohort (n=4221)

**Variable**	**Frequency** (% of whole cohort)
**Age (years)**	
<50 years	1353 (32.1)
≥ 50 years	2868 (67.9)
**Menopausal Status**	
Pre-Menopause	1526 (36.2)
Post-menopause	2695 (63.8)
**Tumour Size (cm) (N=4215)**	
<2	2474 (58.6)
≥2	1741 (41.2)
**Tumour Grade**	
1	757 (17.9)
2	1584 (37.5)
3	1880 (44.5)
**Glandular/tubular differentiation**	
Grade 1: >75% of the tumour forms glands	325 (7.7)
Grade 2: 10-75% of the tumour forms glands	1329 (31.5)
Grade 3: <10% of the tumour forms glands	2567 (60.8)
**Nuclear Pleomorphism**	
Grade 1: Uniform cells with small nuclei	148 (3.5)
Grade 2: large cells with open vesicular nuclei, visible nucleoli. Moderate variability in size and shape	1516 (35.9)
Grade 3: Vesicular nuclei, prominent nucleoli. Marked variation in size and shape	2557 (60.6)
**Mitotic count (per 10 high power fields)**	
<10 mitoses	1864 (44.2)
8-15 mitoses	816 (19.3)
>16 mitoses	1541 (36.5)
**Histological Tumour Type**	
No Special Type (NST)	2591 (61.4)
Invasive Lobular Carcinoma	427 (10.1)
Metaplastic Carcinoma	15 (0.4)
Pure Special Tumour Type (Tubular, Mucinous, Papillary, Micropapillary, Cribriform, ACC)	249 (5.9)
Mixed NST and other tumour types	939 (22.2)
**Lymphovascular invasion**	
Absent	3000 (71.1)
Present	1221 (28.9)
**Lymph node status (N=4220)**	
Negative	2697 (63.9)
Positive	1523 (36.1)
**Nottingham Prognostic Index (N=4214)**	

Good	1522 (36.1)
Moderate	2079 (49.3)
Poor	613 (14.5)
**Cancer stage (N=4220)**	
Lymph node stage 1	2697 (63.9)
Lymph node stage 2	1157 (27.4)
Lymph node stage 3	366 (8.7)
**Oestrogen Receptor Status (N=4105)**	
Negative	961 (22.8)
Positive	3144 (74.5)
**Progesterone Receptor Status (N=3833)**	
Negative	1565 (37.1)
Positive	2268 (53.7)
**HER2 Status (N=3852)**	
Negative	3376 (80.0)
Positive	476 (11.3)
**Molecular Class (N=3281)**	
Luminal A	1317 (31.2)
Luminal B	1142 (27.1)
HER2 Enriched	208 (4.9)
TNBC	614 (14.5)
**Triple negative (N=3966)**	
Non-triple negative	3352 (79.4)
Triple negative	614 (14.5)
**Surgery (N=4221)**	
WLE	2062(48.9)
Mastectomy	2159(51.1)
**Adjuvant Chemotherapy (N=4218)**	
No	3167(75)
Yes	1051(24.9)
**Adjuvant Radiotherapy (N=4173)**	
No	1522(36.1)
Yes	2651(62.8)
**Adjuvant Endocrine therapy (N=4173)**	
No	2108(49.9)
Yes	2065(48.9)

Supplementary Table 3. Antigens, primary antibodies, clone, source, optimal dilution and scoring system used for each immunohistochemical marker.

Antigen	*Antibody*	*Clone*	*Source*	*Antigen Retrieval*	*Dilution / Incubation Time*	*Distribution*	*Scoring system*	*Cut-offs*
**RPA1**	Rabbit Anti-RPA70	ab79398	Abcam	Citrate pH6	1:100 , 1h room temperature	Nuclear	H-score	100
**RPA2**	Mouse Anti-RPA32	ab2175	Abcam	Citrate pH6	1:100 , 1h room temperature	Nuclear	H-score	70
**RPA3**	Rabbit Anti-RPA14	ab97436	Abcam	Citrate pH6	1:50, 1h room temperature	Nuclear cytoplasmic	H-score	Nuclear 50- Cyto 40
**MRE11**	Mouse MAb Anti-MRE11	ab214	Abcam	Citrate pH6	1:800 18hours	Nuclear cytoplasmic	H-score H-score	Low nuclear, Median H-score <90 Low cyto, Median H-score <10
**RAD50**	Mouse MAb Anti-RAD50	ab89	Abcam	Citrate pH6	1:100 18hours	Nuclear	H-score	Low nuclear, Median H-score <100
**NBS1**	Rabbit Anti-NBS1	N3162	Sigma	Citrate pH6	1:100 18hours	Nuclear cytoplasmic	H-score H-score	Low nuclear, Median H-score 590 Low cyto, Median H-score <70
**BRCA1**	BRCA1	MS110	Calbiochem	Citrate pH6	1:100 60 min	Nuclear	% of positive cells	<25% (negative)
**ATM**	Rabbit MAb anti-ATM	Y170	Abcam	Citrate pH6	1:100 18 hours	Nuclear	% of positive cells	<25% (negative)
**XRCC1**	Mouse MAb Anti-XRCC1	33-2-5	Thermo-scientific	Citrate pH6	1:200 20 min	Nuclear	% of positive cells	≥10% (positive)
**Pol β**	Rabbit anti-polβ	Ab26343	Abcam	Citrate pH6	1:200 60 min	Nuclear	H- Score	≥100 (Median H-score, positive)
**BLM**	Rabbit anti BLM	Polyclonal	Novus-Biologicals	Citrate pH6	1:100 18 Hours	Nuclear	H- Score	≥50 (Median H-score, positive)
**RECQL4**	Rabbit Anti RECQL4	Polyclonal	Novus Biologicals	Citrate pH6	1:1000 60 min	Nuclear	H-score	Nuclear ≥215 (Median H-score High)
**CHK2**	Rabbit Anti CHK2	Polyclonal	Abcam	Citrate pH6	1:100 60 min	Nuclear	H- Score	≥100 (Median H-score, positive)
**PARP1**	Mouse MAb Anti-PARP1	7D3-6	BD pharmingen	Citrate pH6	1:1000	Nuclear	% of positive cells	≥10% (positive)
**PR**	Mouse MAb anti-PR	PgR636	Dako-Cytomation	Citrate pH6	1:125 30 min	Nuclear	% positive cells	≥1% positive
**HER2**	Rabbit antihuman c-erbB2	Polyclonal	Dako-Cytomation	None	1:400 60 min	Membrane	See text	See text
**APE1**	Rabbit polyclonal anti-APE1	NB100-101	Novus Biologicals	Citrate pH6	1:500 60 min	Nuclear	H-score	≥100 (positive)

**SMUG1**	Goat anti- SMUG1	Polyclonal	Acris Antibody GmbH	Citrate pH6	1/200<br>15 min	Nuclear	H-score	> 35 (positive)
**pChk1**	Rabbit anti-pChk1	Ab58567	Abcam	Citrate pH6	1:140<br>60 min	Nuclear	H-score	≥50 (High)
**ATR**	Mouse MAb Anti-ATR	1E9	Novus Biologicals	Citrate pH6	1:20<br>18 hours	Nuclear	H-score	≥60 (High)
**BRCA2**	Anti-BRCA2	Polyclonal	Sigma	Citrate pH6	1:200<br>Overnight	Nuclear	H-score	>10
**RECQ5**	Rabbit anti RecqL5	Polyclonal	SigmaAldrich	Citrate pH6	1:100<br>60 min	Nuclear	H - Score	≥10 (positive)
**RECQL1**	Rabbit anti-RECQL1	Polyclonal	Bethyl Laboratories	citrate pH 6.0	1:1,000<br>60 min	Nuclear	H-score	=226
**ERCC1**	Mouse anti-ERCC1	4F9	Dako Ltd	citrate pH 6.0	1:150<br>30 min	Nuclear	H-score	≥ 130
**CHK1**	Rabbit anti-CHK1	Polyclonal	Abcam	citrate pH 6.0	1:150<br>60 min	Nuclear	H-score	>30
**RAD51**	Mouse anti-RAD50	Polyclonal	Abcam	citrate pH 6.0	1:70<br>20 min	Nuclear	H-score	Nuclear ≥8 (High)
**γH2AX**	Ab22551(Phospho S139) Mouse	Monoclonal	Abcam	citrate pH 6.0	1:600<br>1h	Nuclear	H-score	≥ 40
**PIK3CA**	Rabbit anti-PIK3CA antibody	HPA009985	SigmaAldrich	citrate pH 6.0	1:50<br>1h	cytoplasmic	H-score	30 and 100
**FOXA1**	Anti-FOXA1 antibody	ab40868	Abcam	citrate pH 6.0	1:2,000		H-score	100
**FOXO3A**	Forkhead box O3a	Polyclonal (9467)	Cell Signalling Technology	citrate pH 6.0	1:50<br>1h	Nuclear<br>cytoplasmic	H-score	≥10
**P53**	Anti-P53 antibody	D07	Novocastra	citrate pH 6.0	1:50<br>1h	Nuclear	% of positive cells	>10% (negative)
**PIK3CA**	Rabbit anti-PIK3CA antibody	HPA009985	SigmaAldrich	citrate pH 6.0	1:50<br>1h	cytoplasmic	H-score	30 and 100

Supplementary Table 4: RPA1 protein expression and clinicopathological characteristics in DCIS.

RPA1 Expression			
**Variable**	**Low N (%)**	**High N (%)**	**P value**
**DCIS Size (cm)**			0.420
≤2	97(52.7)	87 (47.3)	
>2	122(48.8)	128(51.2)	
**Nuclear Grade**			**0.010**
Low	22(38.6)	35(61.4)	
Moderate	50(42.7)	67(57.3)	
High	148(56.1)	116(43.9)	
**Comedo Necrosis**			0.094
No	68 (44.7)	84 (55.3)	
Yes	152 (53.1)	134 (46.9)	
**Oestrogen Receptor Status**			**0.006**
Negative	61(62.2)	37(37.8)	
Positive	128(46.2)	149(53.8)	
**Progesterone Receptor Status**			**0.001**
Negative	97(59.9)	65(40.1)	
Positive	92(42.2)	126(57.8)	
**HER2 Status**			0.075
Negative	150(48.2)	161(51.8)	
Positive	53(58.9)	37(41.1)	
**Molecular Class**			0.074
Luminal A	82(46.9)	93(53.1)	
Luminal B	27(43.5)	35(56.5)	
HER2 Enriched	31(63.3)	18(36.7)	
TNBC	27(60)	18(40)	

Association with clinical and pathological parameters using categorised data was examined using Chi-squared test. All tests were 2-tailed.

Supplementary Table 5: RPA1 expression and clinicopathological features in invasive breast cancer.

RPA1 Expression			
**Variable**	**Low N (%)**	**High N (%)**	**P value**
**Tumour Size (cm)**			**1.37×10^{-7}**
<2	581 (50.0)	582 (50.0)	
≥2	566 (61.5)	354 (38.5)	
**Tumour Grade**			**2.23×10^{-12}**
1	118 (40.0)	177 (60.0)	
2	376 (50.9)	362 (49.1)	
3	653 (62.2)	397 (37.8)	
**Glandular/tubular differentiation**			**6.10×10^{-7}**
Grade 1: >75% of the tumour forms glands	46 (39.3)	71 (60.7)	
Grade 2: 10-75% of the tumour forms glands	319 (49.5)	325 (50.5)	
Grade 3: <10% of the tumour forms glands	782 (59.2)	540 (40.8)	
**Nuclear Pleomorphism**			**8.56×10^{-7}**
Grade 1: Uniform cells with small nuclei	11 (27.5)	29 (72.5)	
Grade 2: large cells with open vesicular nuclei, visible nucleoli. Moderate variability in size and shape	316 (49.2)	326 (50.8)	
Grade 3: Vesicular nuclei, prominent nucleoli. Marked variation in size and shape	820 (58.5)	581 (41.5)	
**Mitotic count (per 10 high power fields)**			**1.51×10^{-10}**
<10 mitoses	382 (46.3)	443 (53.7)	
8-15 mitoses	227 (57.3)	169 (42.7)	
>16 mitoses	538 (62.4)	324 (37.6)	
**Histological Tumour Type**			**3.00×10^{-6}**
No Special Type (NST)	816 (58.8)	572 (41.2)	
Invasive Lobular Carcinoma	88 (53.3)	77 (46.7)	
Metaplastic Carcinoma	6 (85.7)	1 (14.3)	
Pure Special Tumour Type (Tubular, Mucinous, Papillary, Micropapillary, Cribriform, ACC)	31 (41.3)	44 (58.7)	
Mixed NST and other tumour types	206 (46.0)	242 (54.0)	
**Lymphovascular invasion**			**1.83×10^{-4}**
Absent	735 (52.2)	672 (47.8)	
Present	412 (60.9)	264 (39.1)	
**Lymph node status**			**0.001**
Negative	667 (52.1)	614 (47.9)	
Positive	480 (59.9)	322 (40.1)	
**Nottingham Prognostic Index**			**7.68×10^{-11}**
Good	285 (44.5)	355 (55.5)	
Moderate	637 (58.1)	460 (41.9)	
Poor	225(65)	221(35)	
**Oestrogen Receptor Status**			**4.01×10^{-8}**

Negative	334 (65.6)	175 (34.4)	
Positive	811 (51.7)	758 (48.3)	
**Progesterone Receptor Status**			**9.18×10^{-8}**
Negative	539 (61.8)	333 (38.2)	
Positive	593 (50.0)	594 (50.0)	
**HER2 Status**			0.05
Negative	952 (53.6)	825 (46.4)	
Positive	170 (62.7)	101 (37.3)	
**Ki67 Index**			**2.10×10^{-5}**
Low	353 (48.5)	375 (51.5)	
High	531 (59.1)	368 (40.9)	
**Molecular Class**			**4.77×10^{-9}**
Luminal A	301 (46.4)	345 (53.4)	
HER2 Enriched	84 (70.6)	35 (29.4)	
TNBC	229 (63.8)	130 (36.2)	
Luminal B	379 (56.1)	297 (43.9)	
**Triple negative**			**1.97×10^{-4}**
Non-triple negative	895 (53.0)	793 (47.0)	
Triple negative	229 (68.3)	130 (36.2)	

Association with clinical and pathological parameters using categorised data was examined using Chi-squared test. All tests were 2-tailed

Supplementary Table 6: RPA2 protein expression and clinicopathological characteristics in pure DCIS.

Variable	Low N (%)	High N (%)	P value
**DCIS Size (cm)**			0.520
≤2	61(48)	66(52)	
>2	92(47.7)	101(52.3)	
**Nuclear Grade**			**0.007**
Low	12(36.4)	21(63.6)	
Moderate	32(36.4)	56(63.6)	
High	109(54.5)	91(45.5)	
**Comedo Necrosis**			**0.009**
No	39(37.1)	66(62.9)	
Yes	114(52.8)	102(47.2)	
**Oestrogen Receptor Status**			**0.001**
Negative	46(66.7)	23(33.3)	
Positive	91(42.9)	121(57.1)	
**Progesterone Receptor Status**			**0.003**
Negative	71(58.7)	50(41.3)	
Positive	68(41)	98(59)	
**HER2 Status**			0.294
Negative	108(47.2)	121(52.8)	
Positive	37(54.4)	31(45.6)	
**Molecular Class**			**0.007**
Luminal A	55(43.7)	71(56.3)	
Luminal B	23(45.1)	28(54.9)	
HER2 Enriched	21(58.3)	15(41.7)	
TNBC	23(76.7)	7(23.3)	

Association with clinical and pathological parameters using categorised data was examined using Chi-squared test. All tests were 2-tailed

Supplementary Table 7: RPA2 expression and clinicopathological features in invasive breast cancer.

**Variable**	**Low N (%)**	**High N (%)**	**P value**
**Tumour Size**			**0.001**
<2cm	403 (50.4)	397 (49.6)	
≥2cm	379 (58.9)	265 (41.1)	
**Tumour Grade**			**2.94 x10^{-7}**
1	43 (34.1)	83 (65.9)	
2	273 (51.4)	258 (48.6)	
3	466 (59.2)	321 (40.8)	
**Glandular/tubular differentiation**			
Grade 1: >75% of the tumour forms glands	18 (37.5)	30 (62.5)	**0.012**
Grade 2: 10-75% of the tumour forms glands	208 (51.0)	200 (49.0)	
Grade 3: <10% of the tumour forms glands	556 (56.3)	432 (43.7)	
**Nuclear Pleomorphism**			
Grade 1: Uniform cells with small nuclei	5 (33.3)	10 (66.7)	**1.10 x10^{-5}**
Grade 2: large cells with open vesicular nuclei, visible nucleol. Moderate variability in size and shape	157 (44.1)	199 (55.9)	
Grade 3: Vesicular nuclei, prominent nucleoli. Marked variation in size and shape	620 (57.8)	453 (42.2)	
**Mitotic count**			**2.00 x10^{-5}**
<10 mitoses per 10 high power fields	242 (46.4)	279 (53.6)	
8-15 mitoses per 10 high power fields	161 (54.9)	132 (45.1)	
>16 mitoses per 10 high power fields	379 (60.2)	251 (39.8)	
**Histological Tumour Type**			0.056
No Special Type (NST)	577 (56.1)	451 (43.9)	
Invasive Lobular Carcinoma	48 (46.2)	56 (53.8)	
Metaplastic Carcinoma	3 (100.0)	0 (0.0)	
Pure Special Tumour Type (Tubular, Mucinous, Papillary, Micropapillary, Cribriform, ACC)	13 (52.0)	12 (48.0)	
Mixed NST and other tumour types	141 (49.6)	143 (50.4)	
**Lymphovascular invasion**			**3.80 x10^{-5}**
Absent	487 (50.4)	480 (49.6)	
Present	295 (61.8)	182 (38.2)	
**Lymph node status**			**0.001**
Negative	443 (50.7)	431 (49.3)	
Positive	339 (59.5)	231 (40.5)	
**Nottingham Prognostic Index**			**4.00 x10^{-6}**
Good	172 (44.6)	214 (55.4)	
Moderate	449 (55.7)	357 (44.3)	
Poor	161 (63.9)	91 (36.1)	
**ER Status**			0.229
Negative	208 (56.8)	158 (43.2)	
Positive	573 (53.2)	504 (46.8)	

**PgR Status**			0.083
Negative	352 (56.6)	270 (43.4)	
Positive	420 (52.0)	388 (48.0)	
**HER2 Status**			0.071
Negative	653 (53.2)	574 (46.8)	
Positive	123 (60.0)	82 (40.0)	
**Ki67 Index**			**0.036**
Low	226 (49.0)	235 (51.0)	
High	372 (55.4)	300 (44.6)	
**Molecular Class**			0.052
Luminal A	197 (48.3)	211 (51.7)	
HER2 Enriched	52 (60.5)	34 (39.5)	
TNBC	145 (55.8)	115 (44.2)	
Luminal B	292 (55.7)	232 (44.3)	
**Triple negative**			0.553
Non-triple negative	625 (53.7)	538 (46.3)	
Triple negative	145 (55.8)	115 (44.2)	

Association with clinical and pathological parameters using categorised data was examined using Chi-squared test. All tests were 2-tailed

Supplementary Table 8: RPA3 protein expressions and clinicopathological characteristics in pure DCIS

**Variable**	**RPA3 nuclear expression**			**RPA3 cytoplasm expression**		
	**Low N (%)**	**High N (%)**	**P value**	**Low N (%)**	**High N (%)**	**P value**
**DCIS Size (cm)**			**0.027**			0.073
≤2	50 (45.9)	59 (54.1)		47 (43.1)	62 (56.9)	
>2	101 (59.4)	69 (40.6)		92 (54.1)	78 (45.9)	
**Three Tier Grade**			**0.001**			**0.000037**
Low	12 (35.3)	22(64.7)		9 (26.5)	25(73.5**)**	
Moderate	33 (43.4)	43 (56.6)		28 (36.8)	48(63.2)	
High	107 (62.9)	63 (37.1)		103 (60.6)	67(39.4)	
**Comedo Necrosis**			**0.021**			**0.001**
No	43(44.8)	53 (55.2)		35(36.5)	61(63.5)	
Yes	109(59.2)	75 (40.8)		105(57.1)	79(42.9)	
**Oestrogen Receptor Status**			**0.000079**			**0.000040**
Negative	49 (75.4)	16 (24.6)		46 (70.8)	19 (29.2)	
Positive	86 (47)	97 (53)		76 (41.5)	107 (58.5)	
**Progesterone Receptor Status**			**0.000013**			**5.102 x10^{-7}**
Negative	72 (71.3)	29 (28.7)		69 (68.3)	32 (31.7	
Positive	65 (43.3)	85(56.7)		54(36)	96 (64)	
**HER2 Status**			**0.001**			**0.000189**
Negative	96 (48.2)	103(51.8)		84 (42.2)	115 (57.8)	
Positive	45(72.6)	17(27.4)		43 (69.4)	19 (30.6)	
**Molecular Class**			**0.001**			**0.000073**
Luminal A	54(47.8)	59(52.2)		40(35.4)	73(64.6)	
Luminal B	20(44.4)	25(55.6)		25(55.6)	20(44.4)	
HER2 Enriched	30(83.3)	6(16.7)		22(61.1)	14(38.9)	

TNBC	17(65.4)	9(34.6)		21(80.8)	5(19.2)	

Association with clinical and pathological parameters using categorised data was examined using Chi-squared test. All tests were 2-tailed

Supplementary Table 9: Association between RPA3 protein expression and clinicopathological characteristics in invasive breast cancers.

**Clinicopathological Parameters**	**RPA3 nuclear expression**			**RPA3 cytoplasm expression**		
	**Low N. (%)**	**High N. (%)**	**χ^2 (*p*-value)**	**Low N. (%)**	**High N. (%)**	**χ^2 (*p*-value)**
**Tumour Size (cm)**			**0.000008**			**0.000382**
≤2	463(47.8)	505(52.2)		498 (51.4)	470 (48.6)	
>2	456 (58.5)	323 (41.5)		467 (59.9)	312 (40.1)	
**Tumour Grade**			**$1.99x10^{-10}$**			**$1.72x10^{-8}$**
Low	89 (38.8)	153(63.2)		44(44)	56(56)	
Moderate	326 (49.1)	338(50.9)		253 (46.3)	293(53.7)	
High	504 (59.9)	337(40.1)		668(60.7)	433(39.3)	
**Tumour Stage**			**0.001**			**0.005**
1	525(49.2)	543(50.8)		596(55.8)	472(44.2)	
2	291(56.7)	222(43.3)		261(50.9)	252(49.1)	
3	103(62)	63(38)		108(65.1)	58(34.9)	
**Tubule Formation**			**0.001**			**$1.72x10^{-8}$**
1(>75% definite tubule)	35(35)	65(65)		20(52.6)	18(47)	
2(10%-75% definite tubule)	281(51.5)	265(48.5)		303(54.9)	249(45.1)	
3(<10% definite tubule)	603(54.8)	498(45.2)		642(55.5)	515(44.5)	
**Pleomorphism**			**$3.81x10^{-16}$**			0.923
Grade 1: Uniform cells with small nuclei	6(15.8)	32(84.2)		20(52.6)	18(47.4)	
Grade 2: large cells with open vesicular nuclei, visible nucleoli. Moderate variability in size and shape	228(41.3)	324(58.7)		303(54.9)	249(45.1)	
Grade 3: Vesicular nuclei, prominent nucleoli. Marked variation in size and shape	685(59.2)	472(40.8)		642(55.5)	515(44.5)	
**Mitotic Index**			**$3.187x10^{-9}$**			0.368
M1 (low; mitoses < 10)	314(43.7)	405(56.3)		383(53.3)	336(46.7)	

M2(medium; mitoses 10-18)	203(59.2)	140(40.8)		192(56)	151(44)	
M3(high;mitosis >18)	402(58.7)	283(41.3)		390(56.9)	295(43.1)	
**Tumour Type**			**$2.87x10^{-9}$**			**$4.90\ x10^{-11}$**
Ductal (including mixed)	664 (58.5)	471 (41.5)		630 (55.5)	449 (46.9)	
Lobular	52(36.6)	90(63.4)		115 (81)	15 (19.5)	
Medullary-like	44(48.9)	46(51.1)		52(57.8)	5 (62.5)	
Miscellaneous	23(50)	23(50)		21(45.7)	2 (28.6)	
Special Type	12 (46.2)	14 (53.8)		12(46.2)	16 (64.0)	
Tubular	121 (39.9)	182(60.1)		131(43.2)	172(56.8)	
**Lymphovascular invasion**			**0.00013**			0.565
Nil/Probable	579(49.4)	593(50.6)		653(55.7)	519(44.3)	
Definite	340(59.1)	235(40.9)		312(54.3)	263(45.7)	
**Nottingham Prognostic Index**			**$9.86x10^{-11}$**			**0.017**
Good	224(41.5)	316(58.5)		276(51.1)	264(48.9)	
Moderate	517(55.5)	415(44.5)		520(55.8)	412(44.2)	
Poor	178(64.7)	97(35.3)		169(61.5)	106(38.5)	
**Oestrogen Receptor Status**			**0.001**			**0.018**
Negative	234(60.0)	156(40)		236(60.5)	154(39.5)	
Positive	683(50.5)	669(49.5)		727(53.8)	625(46.2)	
**Progesterone Receptor Status**			**0.000003**			**$2.70x10^{-7}$**
Negative	410(59.8)	276(40.2)		433(63.1)	253(36.9)	
Positive	498(48.2)	535(51.8)		522(50.5)	511(49.5)	
**HER2 Status**			**0.009**			0.061
Negative	766(51.5)	722(48.5)		812(54.6)	676(45.4)	
Positive	142(60.7)	92(39.3)		143(61.1)	91(38.9)	
**Ki67 Index**			**0.004**			0.704
Low	297(47.6)	327(52.4)		335(53.7)	289(46.3)	
High	393(55.4)	316(44.6)		388(54.7)	321(45.3)	
**Molecular Class**			**0.000015**			**0.006**
Luminal A	248(44.8)	305(55.2)		280(50.6)	273(49.4)	

HER2 Enriched	51(60)	34(40)		57(67.1)	28(32.9)	
TNBC	163(60.1)	108(39.9)		163(60.1)	108(39.9)	
Luminal B	328(56.6)	251(43.4)		324(56)	255(44)	
**Triple negative**			**0.006**			0.089
Non-triple negative	734(51.1)	703(48.9)		784(54.6)	653(45.4)	
Triple negative	163(60.1)	108(39,9)		163(60.1)	108(39.9)	

Association with clinical and pathological parameters using categorised data was examined using Chi-squared test. All tests were 2-tailed

Supplementary Table 10: Correlation between RPA1 and expression of other DNA Repair Markers

DNA Repair Marker	Correlation coefficient	P value	Number of samples
RPA2	0.620**	**9.32 x10^{-45}**	405
RPA3_N	0.421**	**3 x10^{-17}**	369
RPA3_C	0.388**	**1.05x10^{-14}**	369
MRE11	0.405**	**3.45 x10^{-17}**	419
RAD50	0.225**	**2.24 x10^{-7}**	456
NBS1	0.134**	**0.006**	592
APE1	-0.009	0.623	351
ATM	0.148**	**0.003**	565
ATR	0.007	0.438	630
BLM	0.186**	**4.4 x10^{-5}**	624
BRCA1	0.343**	**1.09 x10^{-19}**	777
BRCA2	0.039	0.214	767
CHK1	0.268**	**2.38 x10^{-7}**	569
CHK2	0.204**	**3.36 x10^{-7}**	559
DNA-PKcs	0.268**	**3.14 x10^{-13}**	569
POLB	0.321**	**5.14 x10^{-16}**	694
ERCC1	0.202**	**2.8 x10^{-5}**	393
PARP1	0.228**	**2.00 x10^{-6}**	645
pChk1	0.258**	**3.70 x10^{-10}**	786
RAD51	0.322**	**2.06 x10^{-9}**	497
RECQL1	0.321**	**9.36 x10^{-14}**	467
RECQL4	0.155**	**2.77 x 10^{-4}**	510
RECQL5	0.212**	**1.87 x 10^{-4}**	609
SMUG	0.234**	**1.03 x10^{-7}**	607
γH2AX	0.275**	**2.16 x10^{-11}**	541

**. Correlation is significant at the 0.01 level (2-tailed).
*. Correlation is significant at the 0.05 level (2-tailed).

Supplementary Table 11: Correlation between RPA2 and expression of other DNA Repair Markers

DNA Repair Marker	Correlation coefficient	P value	Number of samples
RPA1	0.622**	**$9.32x10^{-45}$**	405
RPA3_N	0.372**	**$4.92x10^{-7}$**	172
RPA3_C	0.202**	**0.008**	172
MRE11	0.172*	**0.0120**	208
RAD50	0.235**	**$1.98\ x10^{-4}$**	226
NBS1	0.043	0.579	300
APE1	0.039	0.524	209
ATM	0.103	0.174	310
ATR	0.051	0.270	339
BLM	0.157**	**0.001**	342
BRCA1	0.324**	**$5.12\ x10^{-13}$**	442
BRCA2	0.093	**0.023**	404
CHK1	0.258**	**0.004**	299
CHK2	0.057	0.126	316
DNA-PKcs	0.182**	**0.002**	312
POLB	0.209**	**$1.5\ x10^{-5}$**	378
ERCC1	0.110	0.084	218
PARP1	0.147**	**0.004**	339
pChk1	0.000	0.187	418
RAD51	0.170**	**0.035**	255
RECQL1	0.148*	0.064	257
RECQL4	0.267**	**$7.29\ x10^{-7}$**	284
RECQL5	0.086	0.421	324
SMUG	0.194**	**0.008**	311
γH2AX	0.142*	**0.008**	300

**. Correlation is significant at the 0.01 level (2-tailed).

Supplementary Table 12: Correlation between RPA3 and expression of other DNA Repair Markers

DNA Repair Marker	Correlation coefficient	P value	Number of samples	Correlation coefficient	P value	Number of samples
	**RPA3 nuclear expression**			**RPA3 cytoplasm expression**		
RPA1	0.421**	**3 x10^{-17}**	369	0.388**	**1.05x10^{-14}**	369
RPA2	0.372**	**4.92x10^{-7}**	172	0.202**	**0.008**	172
MRE11	0.304**	**1.35x10^{-9}**	381	0.256**	**4,24x10^{-7}**	381
RAD50	0.209**	**0.000018**	416	0.190**	**0.000097**	416
NBS1	0.047	0.282	533	-0.055	0.208	533
APE1	-0.007	0.907	258	0.091	0.145	258
ATM	0.035	0.479	414	0.013	0.795	414
ATR	-0.023	0.593	555	0.077	0.069	555
BLM	0.207**	**9.14x10^{-7}**	552	0.033	0.445	552
BRCA1	0.242**	**1.74x10^{-8}**	529	0.147**	**0.001**	529
BRCA2	0.062	0.188	447	0.045	0.343	447
CHK1	0.235**	**3.53x10^{-7}**	458	0.128**	**0.006**	458
CHK2	0.234**	**1.91x10^{-7}**	483	0.203**	**0.000007**	483
DNA-PKcs	0.290**	**5.25x10^{-11}**	493	0.254**	**1.13x10^{-8}**	493
POLB	0.319**	**8.51x10^{-15}**	565	0.264**	**1.72x10^{10}**	565
ERCC1	0.191**	**0.000066**	431	0.109*	**0.023**	431
PARP1	0.221**	**5.11x10^{-7}**	507	0.211**	**0.000002**	507
pChk1	0.324**	**1.84x10^{-16}**	613	0.177**	**0.000011**	613
RAD51	0.320**	**3.96x10^{-11}**	407	0.177**	**0.000328**	407
RECQL1	0.309**	**2.05x10^{-12}**	494	0.269**	**1.27x10^{-9}**	494
RECQL4	0.324**	**1.13x10^{-13}**	499	0.234**	**1.20x10^{-7}**	499
RECQL5	0.220**	**2.18x10^{-7}**	542	0.222**	**1.75x10^{-7}**	542
SMUG	0.150**	**0.001**	470	0.038	0.412	470
γH2AX	0.296**	**1.25x10^{-10}**	455	0.226**	**0.000001**	455

Supplementary Table 13: Correlation between RPA1 and expression of endocrine resistance markers

Endocrine resistance Marker	Correlation coefficient	P value	Number of samples
Ki67	-0.094**	**0.009**	405
VEGF	0.011	0.894	164
PIK3CA	-0.159	0.126	683
MTOR	0.024	0.513	763
CyclinD1	0.111	0.059	290
GATA3	0.221**	**2.16x10^{-7}**	541
FOXA1	0.208**	**3.82x10^{-7}**	588
FOXO3A_C	0.139**	**0.002**	509
FOXO3A_N	0.147**	**0.001**	509
P53	-0.001	0.976	867
ER_beta1	0.120*	**0.032**	322
ER_beta2	0.320**	**1.45x10^{-8}**	300
cMYC_C	0.219**	**0.002**	207
cMYC_N	0.058	0.407	207
pChk1	0.221*	**3.7x10^{-10}**	786
CHK1_C	0.052	0.218	568
CHK1_N	0.209**	**5.27x10^{-7}**	568

**. Correlation is significant at the 0.01 level (2-tailed).

*. Correlation is significant at the 0.05 level (2-tailed).

Supplementary Table 14: Correlation between RPA2 and expression of endocrine resistance markers

**Endocrine resistance Marker**	**Correlation coefficient**	**P value**	**Number of samples**
Ki67	-0.025	0.617	416
VEGF	0.137	0.221	82
PIK3CA	0.007	0.891	357
MTOR	0.020	0.687	401
CyclinD1	0.005	0.954	120
GATA3	0.122*	**0.035**	299
FOXA1	0.024	0.671	311
FOXO3A_C	0.156**	**0.009**	279
FOXO3A_N	0.087	0.087	279
P53	0.054	0.246	457
ER_beta1	0.087	0.329	128
ER_beta2	0.228*	**0.011**	124
cMYC_C	0.194	0.061	94
cMYC_N	0.139	0.182	94
CHK1_C	0.159**	**0.006**	298
CHK1_N	0.150**	**0.010**	289

****. Correlation is significant at the 0.01 level (2-tailed).**
***. Correlation is significant at the 0.05 level (2-tailed).**

Supplementary Table 15: Correlation between RPA3 and expression of endocrine resistance marker

Endocrine resistance Marker	Correlation coefficient	P value	Number of samples	Correlation coefficient	P value	Number of samples
	**RPA3 nuclear expression**			**RPA3 cytoplasm expression**		
Ki67	0.020	0.632	548	0.025	0.560	548
VEGF	0.092	0.307	125	0.175	0.051	125
PIK3CA	-0.158**	**0.000326**	516	-.057	0.197	516
MTOR	0.043	0.302	575	0.117**	**0.005**	575
CyclinD1	-0.061	0.229	295	0.080	0.172	295
GATA3	0.150**	**0.002**	413	0.115*	**0.020**	413
FOXA1	0.237**	**3.16×10^{-7}**	456	0.114*	**0.015**	456
FOXO3A_C	-0.018	0.725	373	0.100	0.054	373
FOXO3A_N	0.143**	**0.006**	373	-.0.048	0.353	373
P53	-0.014	0.730	634	-0.028	0.478	634
ER_beta1	0.026	0.648	310	0.126*	**0.026**	310
ER_beta2	0.265**	**0.000007**	280	0.188**	**0.002**	280
cMYC_C	0.181*	**0.012**	191	0.154*	**0.034**	191
cMYC_N	0.240**	**0.001**	191	0.023	0.756	191
CHK1_C	0.084	0.071	458	0.244**	**1.29×10^{-7}**	458
CHK1_N	0.235**	**3.53×10^{-7}**	458	0.128**	**0.006**	458

Supplementary Table 16: Pathway analysis of genes differentially expressed between tumours with low and high RPA complex.

Gene Set	Description	Size	Expect	Ratio	P Value	FDR
Genes expressed higher in low RPA tumours						
hsa04740	Olfactory transduction	448	22.613	3.7147	0	0
hsa05034	Alcoholism	180	9.0854	4.2926	2.9976e-15	4.8861e-13
hsa05322	Systemic lupus erythematosus	133	6.7131	4.9158	7.1054e-15	7.7212e-13
hsa04742	Taste transduction	83	4.1894	4.2966	1.0902e-7	0.0000088851
hsa00140	Steroid hormone biosynthesis	60	3.0285	4.2926	0.0000066300	0.00043228
hsa00980	Metabolism of xenobiotics by cytochrome P450	76	3.8361	3.6496	0.000021370	0.0011611
hsa05204	Chemical carcinogenesis	82	4.1389	3.3825	0.000051856	0.0024150
hsa00982	Drug metabolism	72	3.6342	3.3020	0.00022474	0.0091580
hsa00830	Retinol metabolism	67	3.3818	3.2527	0.00046723	0.016924
hsa04080	Neuroactive ligand-receptor interaction	277	13.981	1.9311	0.00072386	0.023598
Genes expressed lower in low RPA tumours						
hsa04080	Neuroactive ligand-receptor interaction	277	1.7143	5.2498	0.000039338	0.012824

SUPPLEMENTARY MATERIALS

Supplementary Figures

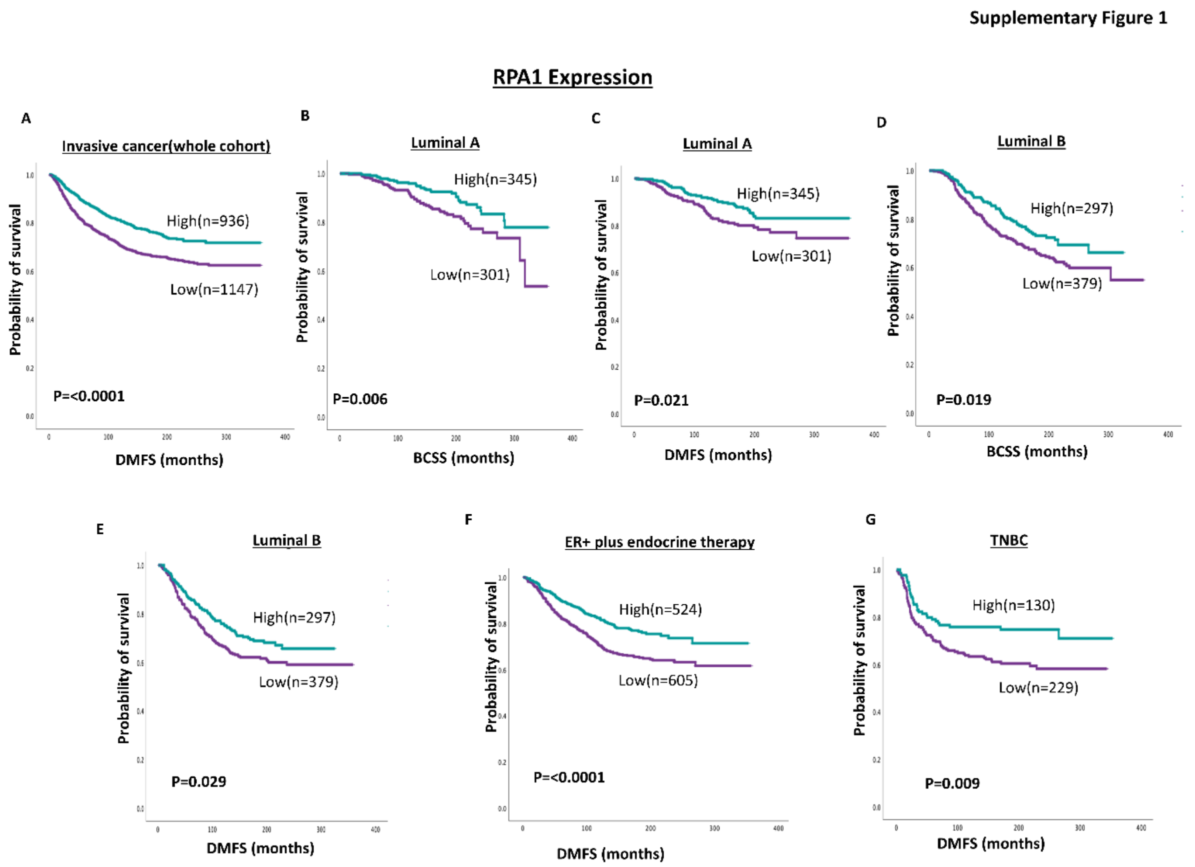

Supplementary Figure 1: RPA1 and breast cancer. (A). Kaplan-Meier curve for RPA1 nuclear protein expression and DMFS in in whole cohort. **(B).** Kaplan-Meier curve for RPA1 nuclear protein expression and BCSS in Luminal A cohort. (**C**). Kaplan-Meier curve for RPA1 nuclear protein expression and DMFS in Luminal A cohort. **(D).** Kaplan-Meier curve for RPA1 nuclear protein expression and BCSS in Luminal B cohort. (**E**). Kaplan-Meier curve for RPA1 nuclear protein expression and DMFS in Luminal B cohort. **(F).** Kaplan-Meier curve for RPA1 nuclear protein expression and DMFS in TNBC cohort. Survival rates were determined using Kaplan–Meier method and compared by the log-rank test. All analyses were conducted using Statistical Package for the Social Sciences (SPSS, version 22, Chicago, IL, USA) software for windows. P value of less than 0.05 was identified as statistically significant.

Supplementary Figure 2

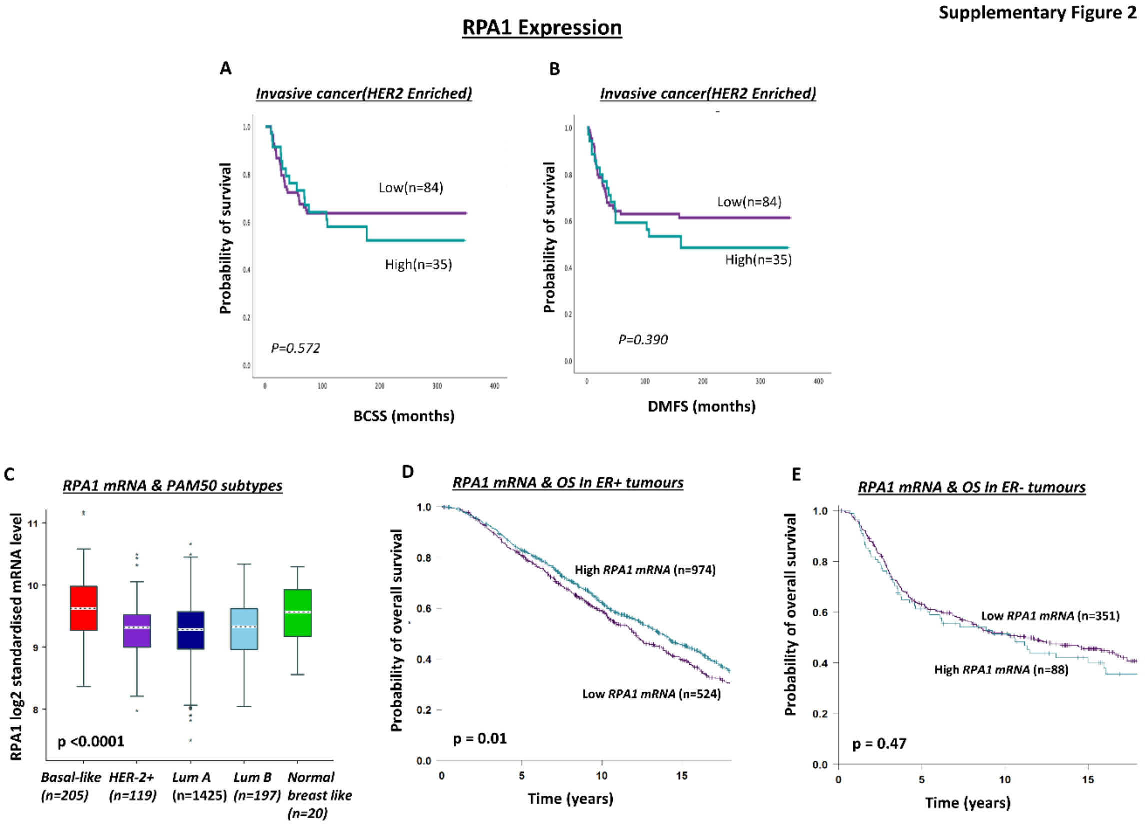

Supplementary Figure 2: RPA1 and breast cancer. **(A).** Kaplan-Meier curve for RPA1 nuclear protein expression and BCSS in HER2 Enriched cohort. (**B**). Kaplan-Meier curve for RPA1 nuclear protein expression and DMFS in HER2 Enriched cohort. (C). *RPA1 mRNA* expression and PAM50 molecular subtypes of breast cancer [The white dotted line represents the median, upper closed bar represents the upper quartile value, closed lower bar represents the lower quartile value, upper grey line represents maximum data value, lower line represents minimum data value, dots are outliers.]. **(D).** Kaplan–Meier curve for RPA1 mRNA expression and BCSS in ER+ cohort. **(E)** Kaplan–Meier curve for RPA2 mRNA expression and BCSS in ER− cohort. Survival rates were determined using Kaplan–Meier method and compared by the log-rank test. All analyses were conducted using Statistical Package for the Social Sciences (SPSS, version 22, Chicago, IL, USA) software for windows. P value of less than 0.05 was identified as statistically significant.

Supplementary Figure 3

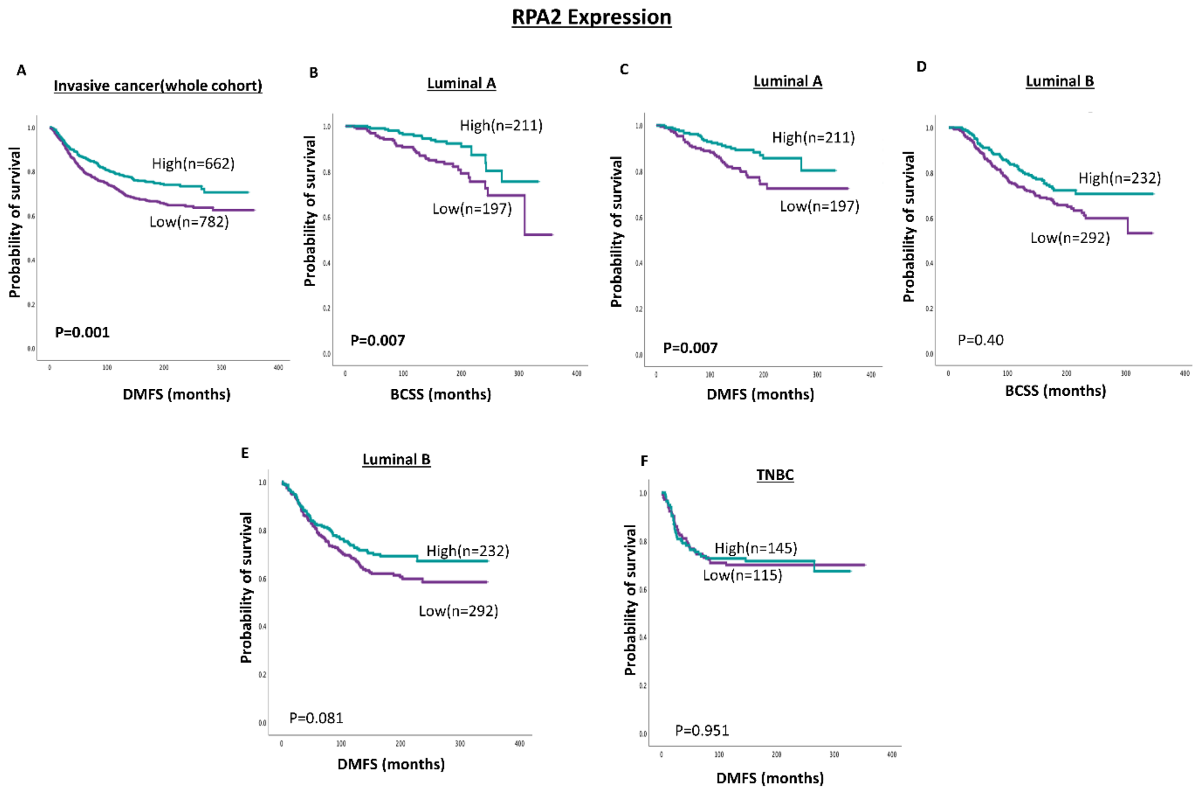

Supplementary Figure 3: RPA2 and breast cancer. **(A).** Kaplan-Meier curve for RPA2 nuclear protein expression and DMFS in in whole cohort. **(B).** Kaplan-Meier curve for RPA2 nuclear protein expression and BCSS in Luminal A cohort. (**C**). Kaplan-Meier curve for RPA2 nuclear protein expression and DMFS in Luminal A cohort. **(D).** Kaplan-Meier curve for RPA2 nuclear protein expression and BCSS in Luminal B cohort. (**E**). Kaplan-Meier curve for RPA2 nuclear protein expression and DMFS in Luminal B cohort. **(F).** Kaplan-Meier curve for RPA2 nuclear protein expression and DMFS in TNBC cohort. Survival rates were determined using Kaplan–Meier method and compared by the log-rank test. All analyses were conducted using Statistical Package for the Social Sciences (SPSS, version 22, Chicago, IL, USA) software for windows. P value of less than 0.05 was identified as statistically significant.

RPA2 Expression **Supplementary Figure 4**

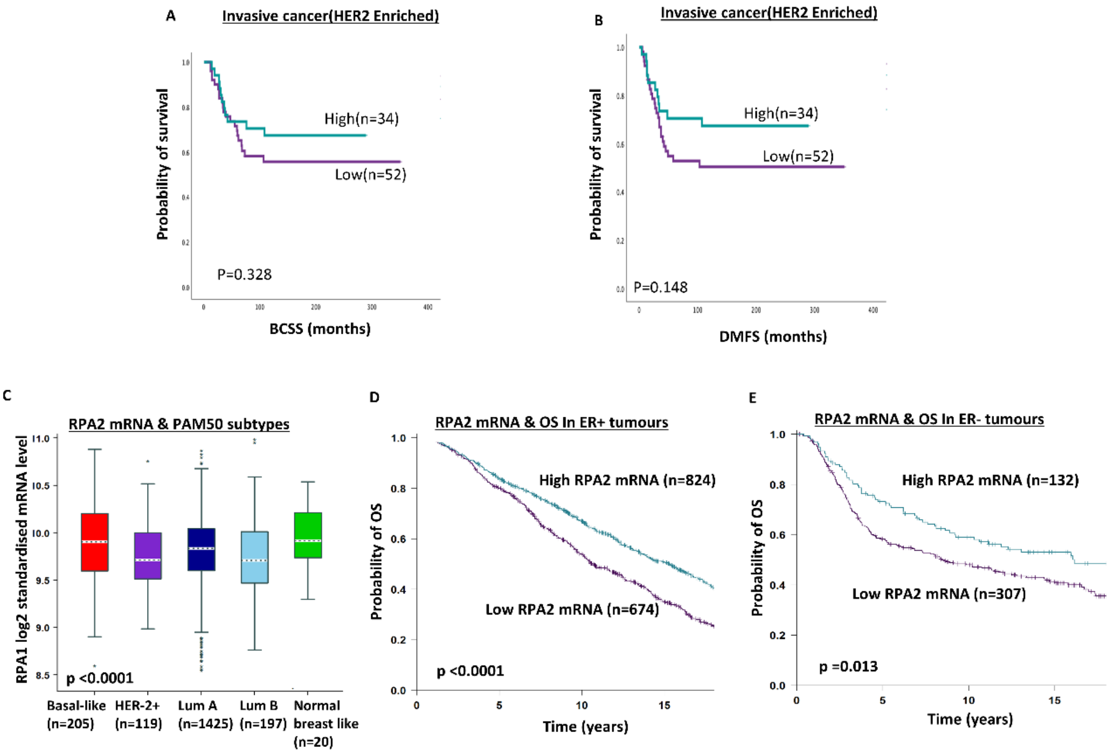

Supplementary Figure 4: RPA2 and breast cancer. (A). Kaplan-Meier curve for RPA2 nuclear protein expression and BCSS in HER2 Enriched cohort. (**B**). Kaplan-Meier curve for RPA2 nuclear protein expression and DMFS in HER2 Enriched cohort. **(C).** *RPA2 mRNA* expression and PAM50 molecular subtypes of breast cancer [The white dotted line represents the median, upper closed bar represents the upper quartile value, closed lower bar represents the lower quartile value, upper grey line represents maximum data value, lower line represents minimum data value, dots are outliers.]. **(D).** Kaplan–Meier curve for RPA2 mRNA expression and BCSS in ER+ cohort. **(E).** Kaplan–Meier curve for RPA2 mRNA expression and BCSS in ER− cohort. Survival rates were determined using Kaplan–Meier method and compared by the log-rank test. All analyses were conducted using Statistical Package for the Social Sciences (SPSS, version 22, Chicago, IL, USA) software for windows. P value of less than 0.05 was identified as statistically significant.

Supplementary Figure 5

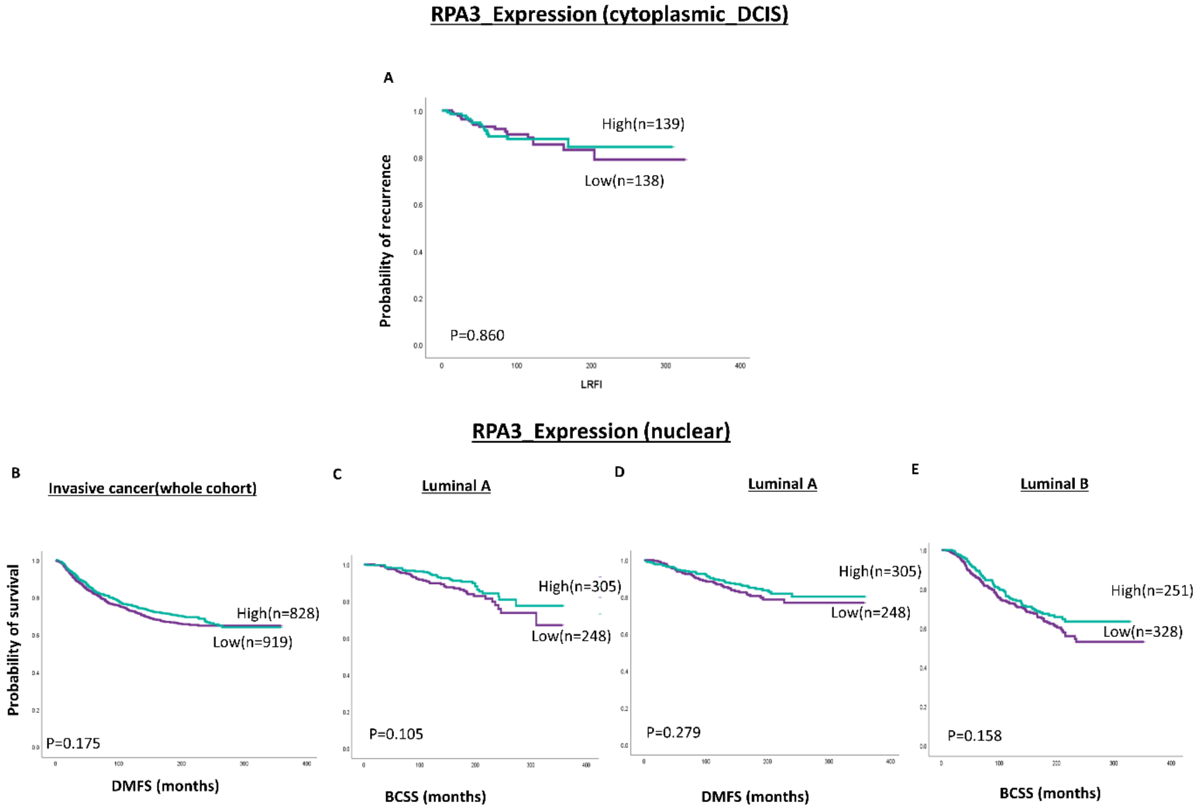

Supplementary Figure 5: RPA3 and breast cancer. **(A).** Kaplan-Meier curve for RPA3 cytplasmic protein expression and LRFI in DCIS cohort. **(B).** Kaplan-Meier curve for RPA3 nuclear protein expression and DMFS in in whole cohort. **(C).** Kaplan-Meier curve for RPA3 nuclear protein expression and BCSS in Luminal A cohort. (**D**). Kaplan-Meier curve for RPA3 nuclear protein expression and DMFS in Luminal A cohort. **(E).** Kaplan-Meier curve for RPA3 nuclear protein expression and BCSS in Luminal B cohort. Survival rates were determined using Kaplan–Meier method and compared by the log-rank test. All analyses were conducted using Statistical Package for the Social Sciences (SPSS, version 22, Chicago, IL, USA) software for windows. P value of less than 0.05 was identified as statistically significant.

Supplementary Figure 6

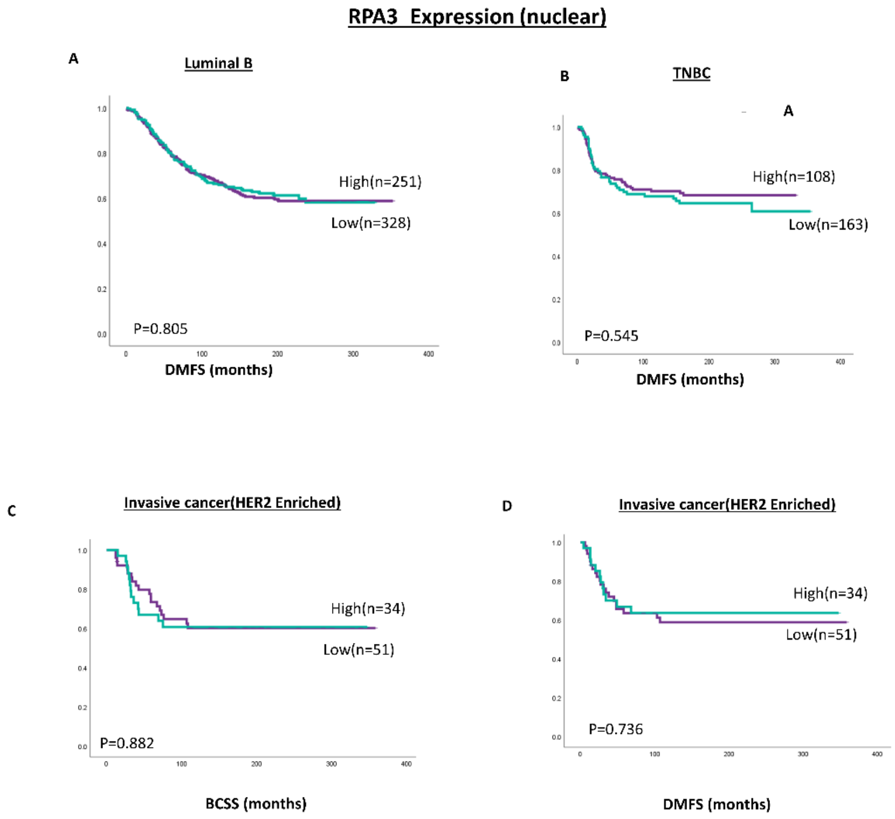

Supplementary Figure 6: RPA3 and breast cancer. (**A**). Kaplan-Meier curve for RPA3 nuclear protein expression and DMFS in Luminal B cohort. **(B)**. Kaplan-Meier curve for RPA3 nuclear protein expression and DMFS in TNBC cohort. **(C).** Kaplan-Meier curve for RPA3 nuclear protein expression and BCSS in HER2 Enriched cohort. (**D)**. Kaplan-Meier curve for RPA3 nuclear protein expression and DMFS in HER2 Enriched cohort. Survival rates were determined using Kaplan–Meier method and compared by the log-rank test. All analyses were conducted using Statistical Package for the Social Sciences (SPSS, version 22, Chicago, IL, USA) software for windows. P value of less than 0.05 was identified as statistically significant.

Supplementary Figure 7

RPA3_Expression (cytoplasmic)

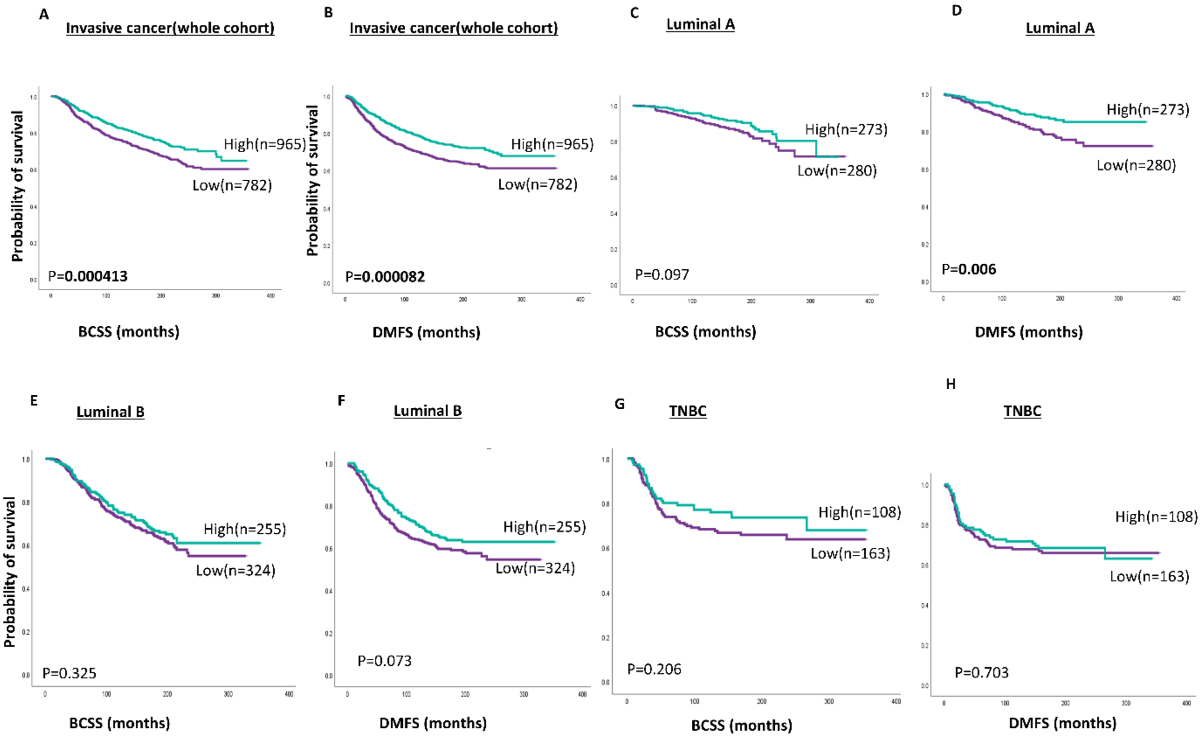

Supplementary Figure 7: RPA3 and breast cancer. **(A)**. Kaplan-Meier curve for RPA3 cytoplasmic protein expression and breast cancer specific survival (BCSS) in whole cohort**.** **(B).** Kaplan-Meier curve for RPA3 cytoplasmic protein expression and DMFS in in whole cohort. **(C).** Kaplan-Meier curve for RPA3 cytoplasmic protein expression and BCSS in Luminal A cohort. (**D**). Kaplan-Meier curve for RPA3 cytoplasmic protein expression and DMFS in Luminal A cohort. **(E).** Kaplan-Meier curve for RPA3 cytoplasmic protein expression and BCSS in Luminal B cohort. (**F**). Kaplan-Meier curve for RPA3 cytoplasmic protein expression and DMFS in Luminal B cohort. **(G).** Kaplan-Meier curve for RPA3 cytoplasmic protein expression and BCSS in TNBC cohort. (**H**). Kaplan-Meier curve for RPA3 cytoplasmic protein expression and DMFS in TNBC cohort. Survival rates were determined using Kaplan–Meier method and compared by the log-rank test. All analyses were conducted using Statistical Package for the Social Sciences (SPSS, version 22, Chicago, IL, USA) software for windows. P value of less than 0.05 was identified as statistically significant.

Supplementary Figure 8

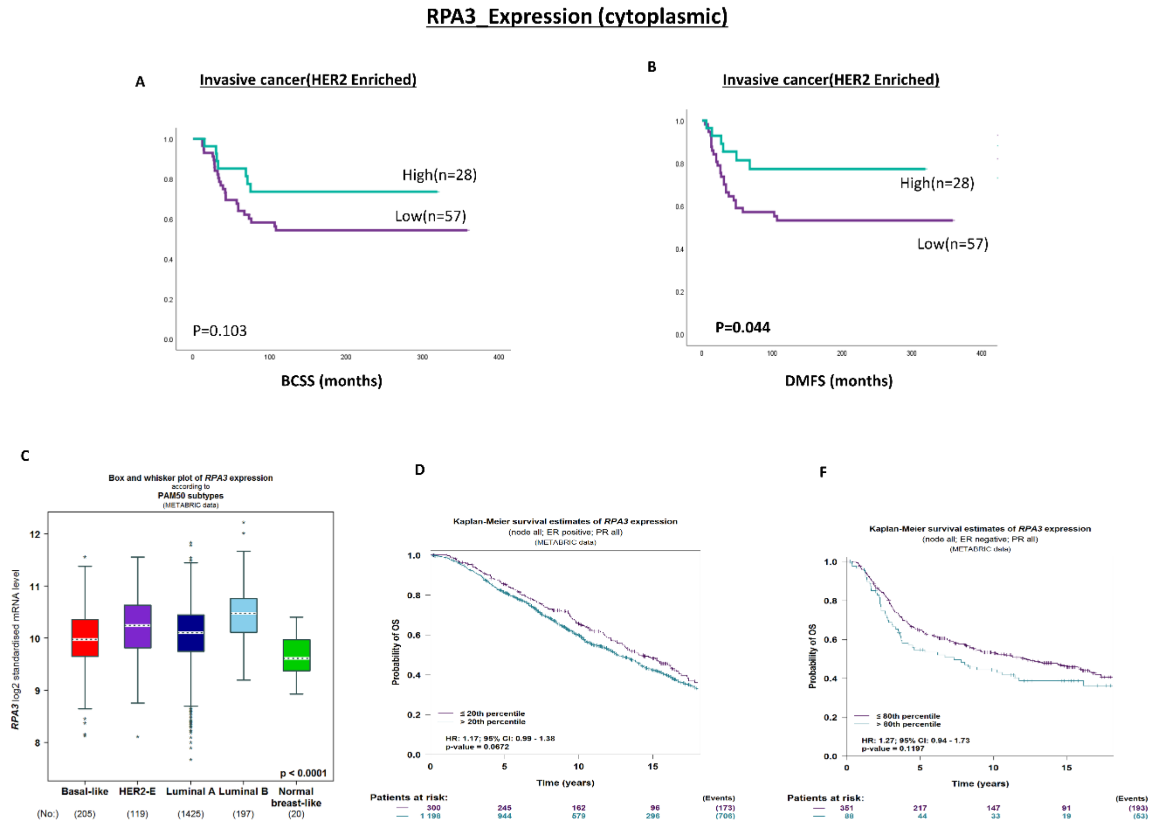

Supplementary Figure 8: RPA3 and breast cancer. **(A).** Kaplan-Meier curve for RPA3 cytoplasmic protein expression and BCSS in HER2 Enriched cohort. (**B)**. Kaplan-Meier curve for RPA3 cytoplasmic protein expression and DMFS in HER2 Enriched cohort. **(C).** *RPA3 mRNA* expression and PAM50 molecular subtypes of breast cancer [The white dotted line represents the median, upper closed bar represents the upper quartile value, closed lower bar represents the lower quartile value, upper grey line represents maximum data value, lower line represents minimum data value, dots are outliers.]. **(D).** Kaplan–Meier curve for *RPA3 mRNA* expression and BCSS in ER+ cohort. **(E).** Kaplan–Meier curve for *RPA3 mRNA* expression and BCSS in ER− cohort. Survival rates were determined using Kaplan–Meier method and compared by the log-rank test. All analyses were conducted using Statistical Package for the Social Sciences (SPSS, version 22, Chicago, IL, USA) software for windows. P value of less than 0.05 was identified as statistically significant.

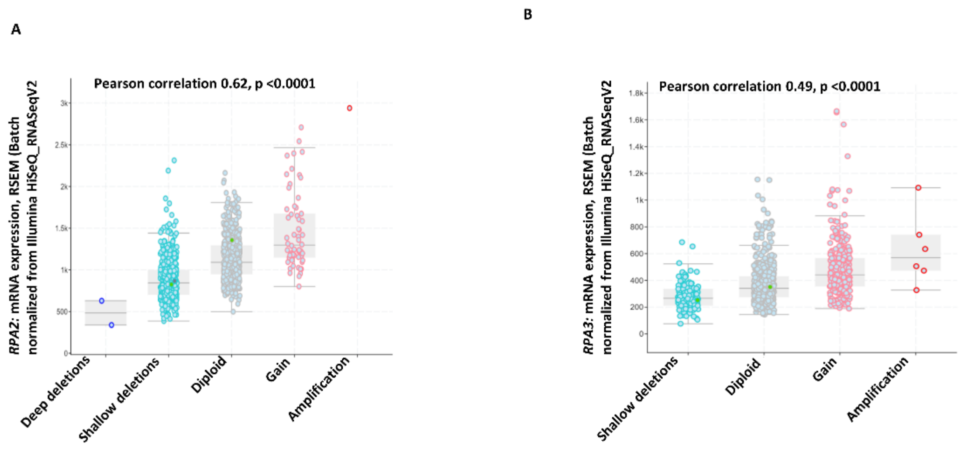

Supplementary Figure 9: RPA2 and RPA3 bioinformatics. **(A).** Comparison of RPA2 gene expression to copy number variation in TCGA-BRCA Pan cancer cohort (n = 994). GISTIC analysis is shown for changes in *RPA2* mRNA levels in tumours with copy number variations for TCGA-BRCA Pan cancer cohort (n = 994). The expression data was from normalized illumina HiSeq RNA-Seq data. The copy number variations are deep deletions (>2 copies deleted), shallow deletion (few copies altered), diploid, gains (few copies gained), amplification (>2 copies gained). (**B**). Comparison of RPA3 gene expression to copy number variation in TCGA-BRCA Pan cancer cohort (n = 994) is shown here. [The grey line represents the median, upper closed bar represents the upper quartile value, closed lower bar represents the lower quartile value, upper grey line represents maximum data value, lower line represents minimum data value, dots represent individual values]

Supplementary Figure 10

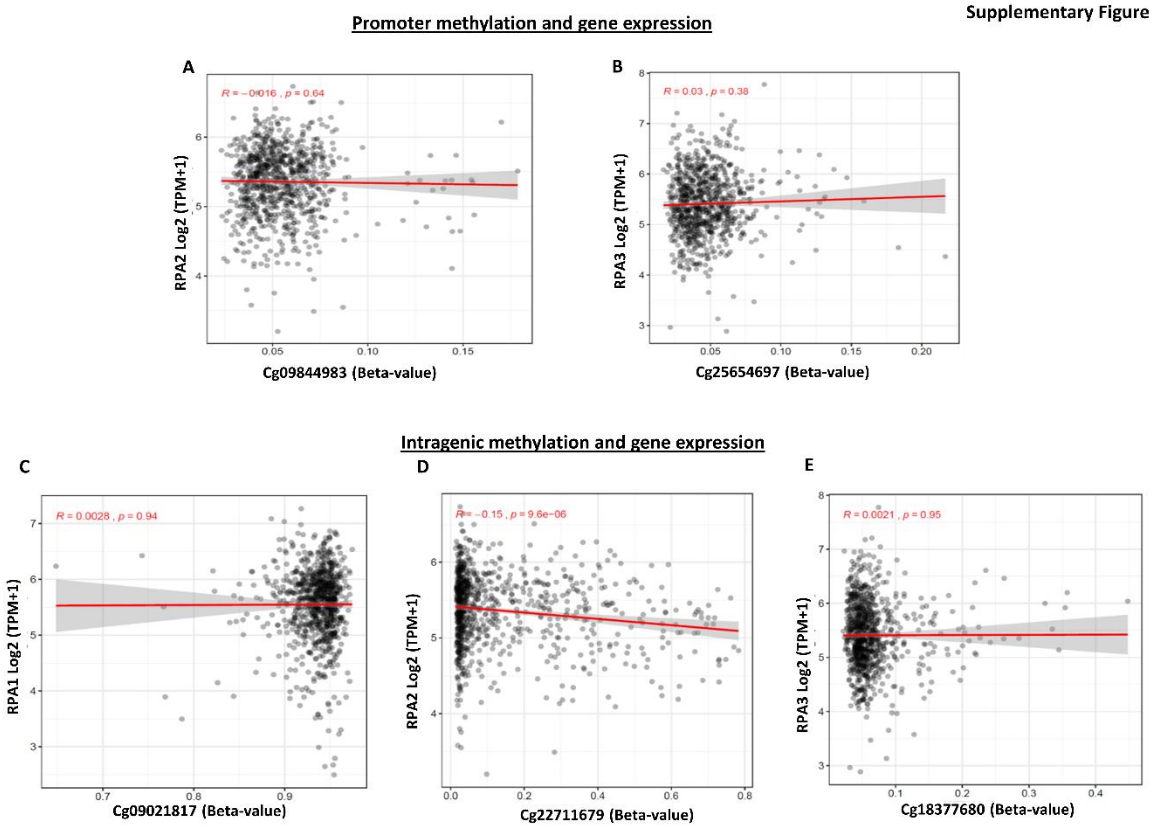

Supplementary Figure 10: RPA bioinformatics. (**A**). DNA promoter methylation correlations with RPA2 gene expression is shown here. (**B**) DNA promoter methylation correlations with RPA3 gene expression is shown here. Intragenic methylation and correlation to RPA1 (**C**), RPA2 (**D**) and RPA3 (**E**) gene expression is shown here (see methods sections for more details**)**.

Supplementary Figure 11

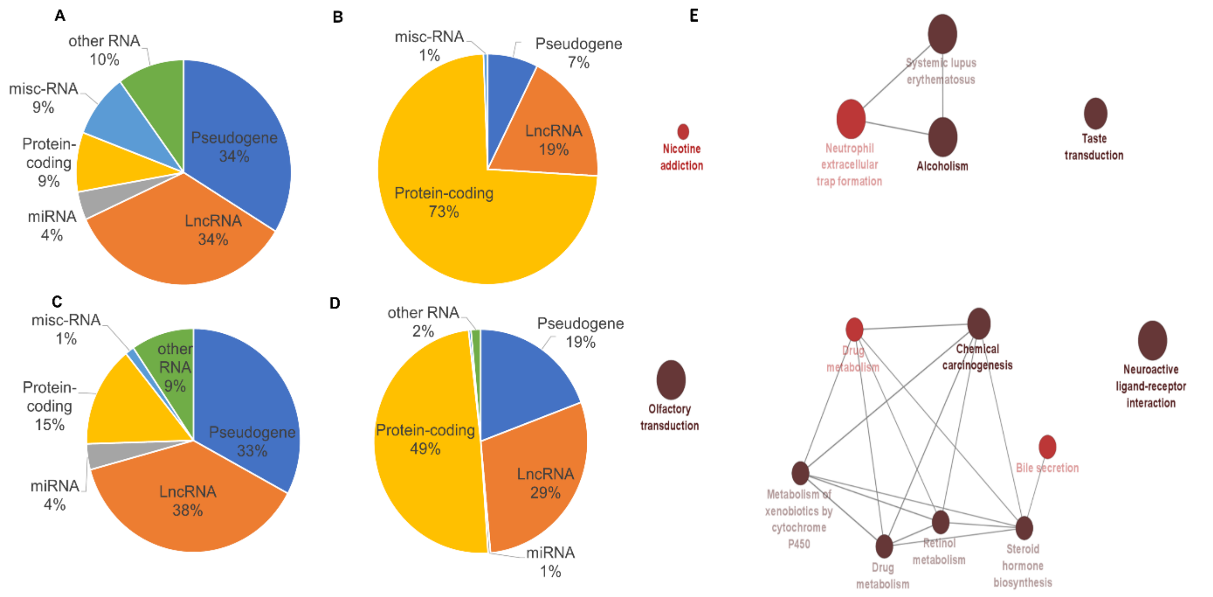

Supplementary Figure 11: RPA bioinformatics. The percentage of non-coding RNAs (lncRNA, pseudogenes and miRNAs) plus coding genes are shown for **A**) RNAs with high expression in low *RPA2* tumours (n = 8737 confirmed gene types), **B**) RNAs with low expression in low *RPA2* tumours (n = 162), **C**) RNAs with high expression in low *RPA3* tumours (n = 8581), **D**) RNAs with low expression in low *RPA3* tumours (n = 274) and **(E)** Cluego analysis was performed to identify common genes and pathways that were differentially expressed between low and high RPA. Genes (938) were represented in 14 terms and 6 groups (term-term interaction).

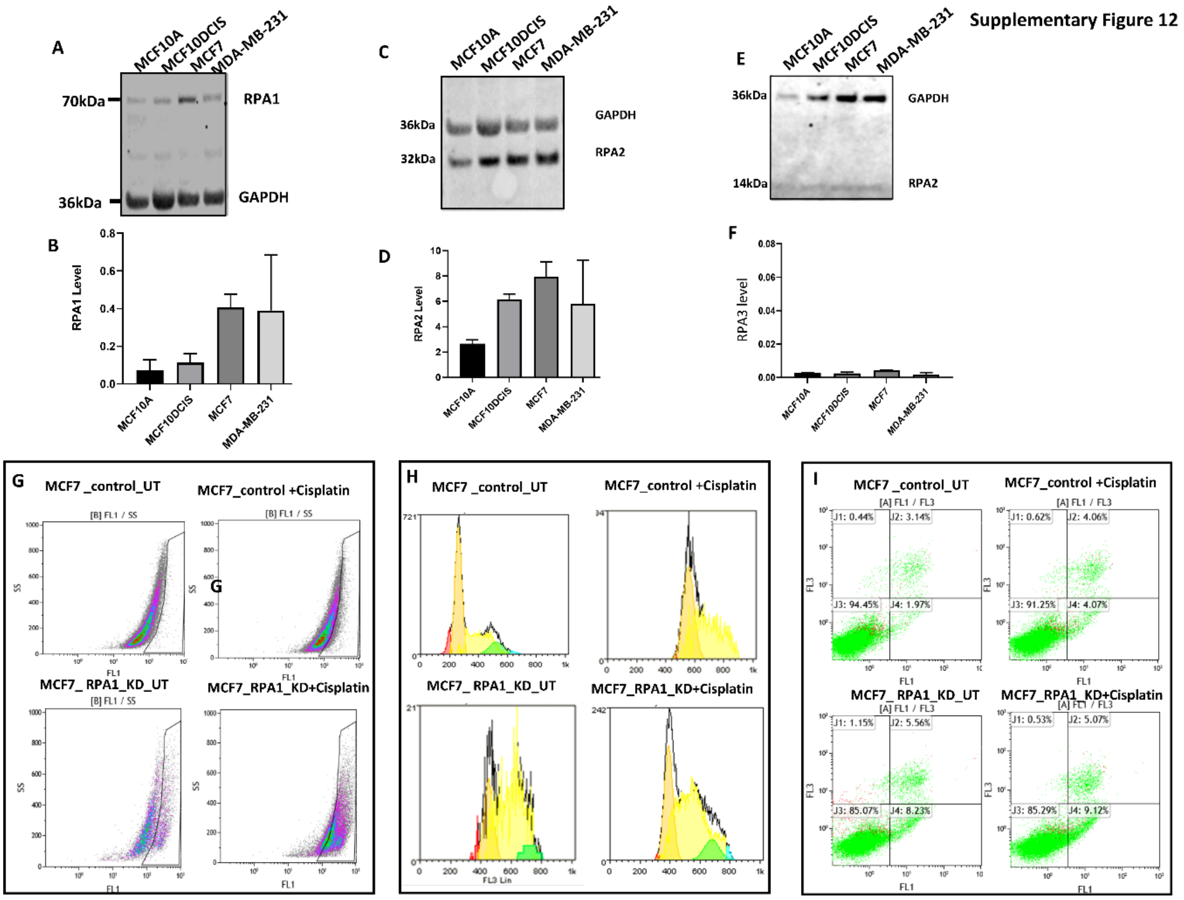

Supplementary Figure 12: RPA expression in a panel of breast cell lines. (A). Western blot of RPA1 protein expression in MCF10A, MCF10DCIS, MCF7 and MDA-MB-231 cells. **(B)**. Quantification of RPA1 expression in MCF10A, MCF10DCIS, MCF7 and MDA-MB-231 cells. **(C).** Western blot of RPA2 protein expression in MCF10A, MCF10DCIS, MCF7 and MDA-MB-231 cells. **(D).** Quantification of RPA2 expression in MCF10A, MCF10DCIS, MCF7 and MDA-MB-231 cells. **(E).** Western blot of RPA3 protein expression in MCF10A, MCF10DCIS, MCF7 and MDA-MB-231 cells. **(F).** Quantification of RPA3 expression in MCF10A, MCF10DCIS, MCF7 and MDA-MB-231 cells. **(G).** Gamma H2AX analysis by flow cytometry in MCF7 control and RPA1_KD cells untreated or treated with cisplatin. **(H).** Cell cycle analysis by flow cytometry in MCF7 control and RPA1_KD cells untreated or treated with cisplatin. **(I).** Annexin V assay analysis by flow cytometry in MCF7 control and RPA1_KD cells untreated or treated with cisplatin.

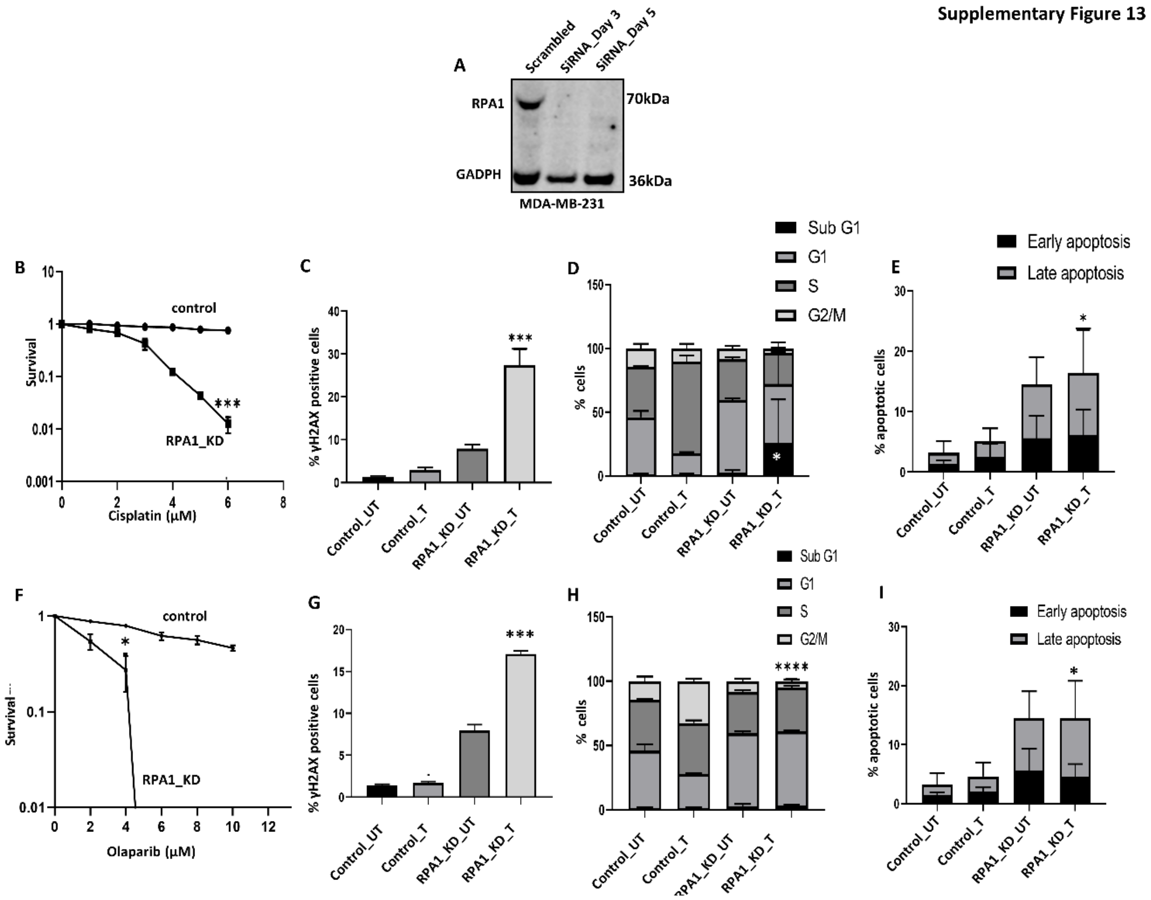

Supplementary Figure 13: RPA1 depletion and cisplatin and olaparib sensitivity in breast cancer cells. (**A**) RPA1 siRNA knock down in MDA-MB-231 cells. Lysates were collected at day3 and day5. (**B**) Clonogenic survival assay for cisplatin sensitivity in MDA-MB-231 cells control and MCF7_RPA1_KD cells. **(C)** Quantification of γH2AX positive cells by flow cytometry. **(D)** Cell cycle analysis by flow cytometry. **(E)** AnnexinV analysis for apoptotic cells in MDA-MB-231 control and RPA1_knock down cells treated with 5 µM cisplatin for 24hrs. (**F**) Clonogenic survival assay for Olaparib sensitivity in MDA-MB-231 cells control and MDA-MB-231_RPA1_KD cells. (**G**) Quantification of γH2AX positive cells by flow cytometry. **(H)** Cell cycle analysis by flow cytometry. **(I)** AnnexinV analysis for apoptotic cells in MDA-MB-231 control and RPA1_knock down cells treated with 6µM Olaparib for 24 hrs. Statistical analysis was conducted as on GraphPad Prism7 software. To compare between two groups, student- T-tests analysis was performed. One-way ANOVA was performed to compare between more than two groups (variances analyses). Two-way ANOVA was used to analyse

two variables such as Annexin V analysis and cell cycle analysis. All experiments were expressed as means ± standard deviation S.D. of three independent experiments. Error bars represent standard error of mean between experiments. UN = untreated, T = treated. ‘*’ = p value <0.05, ‘**’ = p value <0.001, ‘***’ = p value <0.0001.

Supplementary Figure 14

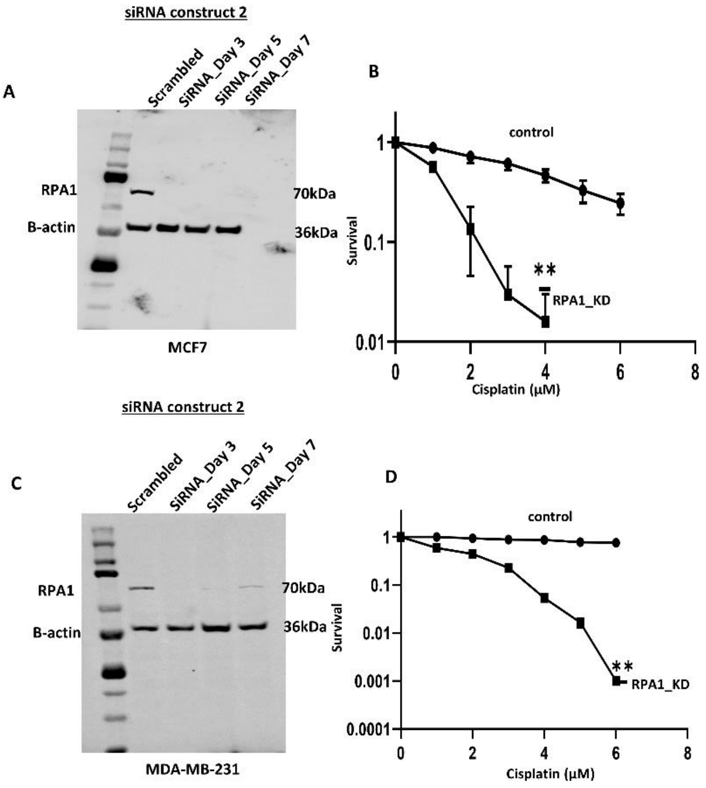

Supplementary Figure 14: RPA depletion and cisplatin sensitization. (A). RPA1 siRNA construct 2 knock down in MCF7 cells. Lysates were collected at day3 and day5. **(B).** Clonogenic survival assay for cisplatin sensitivity in MCF7 cells control and MCF7_RPA1_KD cells. **(C).** RPA1 siRNA construct 2 knock down in MDA-MB-231 cells. Lysates were collected at day3 and day5. (**D**) Clonogenic survival assay for cisplatin sensitivity in MDA-MB-231 cells control and MCF7_RPA1_KD cells. Statistical analysis was conducted as on GraphPad Prism7 software. To compare between two groups, student- T-tests analysis was performed. One-way ANOVA was performed to compare between more than two groups (variances analyses). Two-way ANOVA was used to analyse two variables such as Annexin V analysis and cell cycle analysis. All experiments were expressed as means ± standard deviation S.D. of three independent experiments. Error bars represent standard error of mean between experiments. UN = untreated, T = treated. '*' = p value <0.05, '**' = p value <0.001, '***' = p value <0.0001.

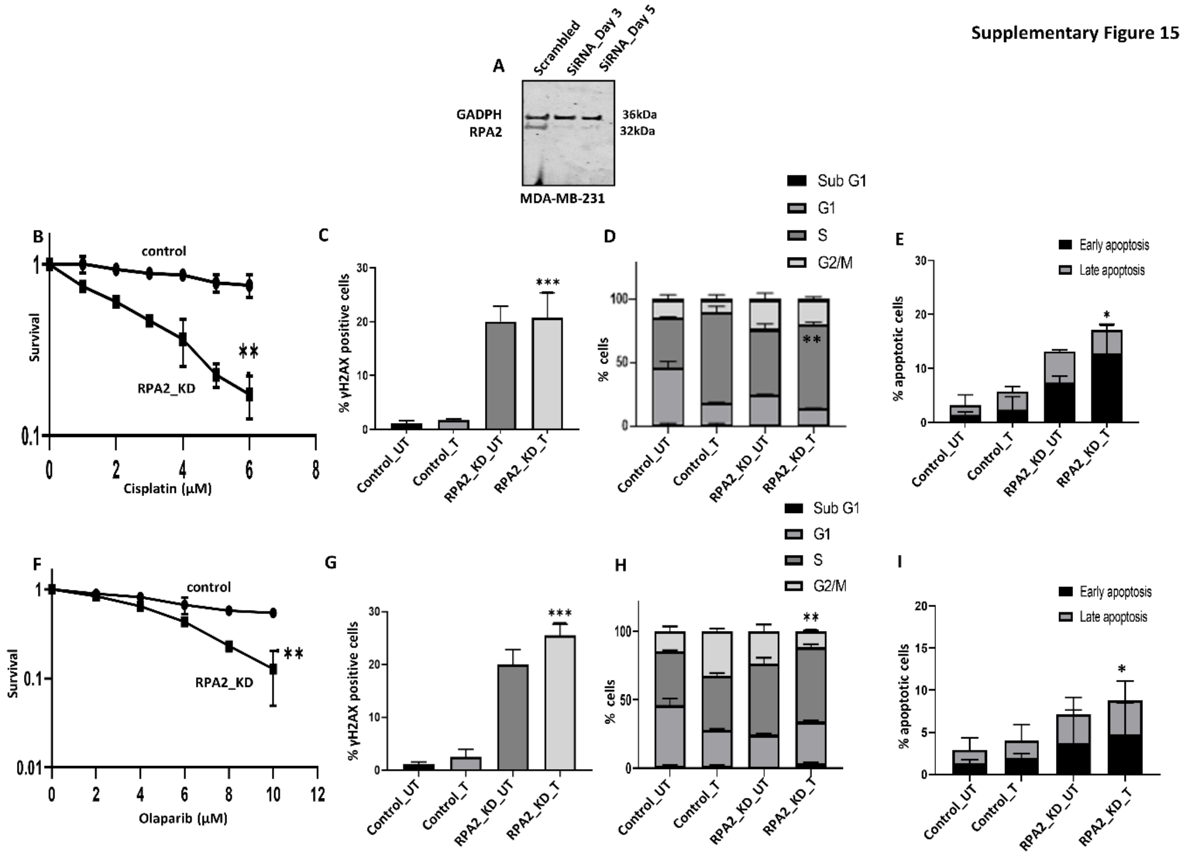

Supplementary Figure 15: RPA2 depletion and cisplatin and Olaparib sensitivity in breast cancer cells. (**A**) RPA2 siRNA knock down in MDA-MB-231 cells. Lysates were collected at day3 and day5. (**B**) Clonogenic survival assay for cisplatin sensitivity in MDA-MB-231 cells control and MCF7_RPA2_KD cells. **(C)** Quantification of γH2AX positive cells by flow cytometry. **(D)** Cell cycle analysis by flow cytometry. **(E)** AnnexinV analysis for apoptotic cells in MDA-MB-231 control and RPA2_knock down cells treated with 5 μM cisplatin for 24hrs. (**F**) Clonogenic survival assay for Olaparib sensitivity in MDA-MB-231 cells control and MDA-MB-231_RPA2_KD cells. (**G**) Quantification of γH2AX positive cells by flow cytometry. **(H)** Cell cycle analysis by flow cytometry. **(I)** AnnexinV analysis for apoptotic cells in MDA-MB-231 control and RPA2_knock down cells treated with 6μM Olaparib for 24 hrs. Statistical analysis was conducted as on GraphPad Prism7 software. To compare between two groups, student- T-tests analysis was performed. One-way ANOVA was

performed to compare between more than two groups (variances analyses). Two-way ANOVA was used to analyse two variables such as Annexin V analysis and cell cycle analysis. All experiments were expressed as means ± standard deviation S.D. of three independent experiments. Error bars represent standard error of mean between experiments. UN = untreated, T = treated. ‘*’ = p value <0.05, ‘**’ = p value <0.001, ‘***’ = p value <0.0001.

Supplementary Figure S16

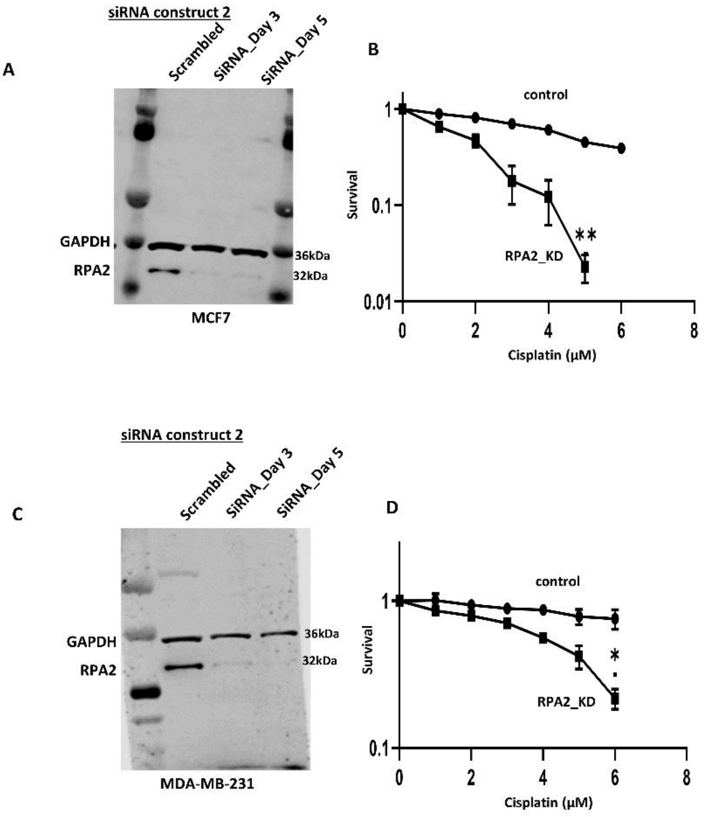

Supplementary Figure 16: RPA2 depletion and cisplatin sensitization. (A.) RPA2 siRNA construct 2 knock down in MCF7 cells. Lysates were collected at day3 and day5. **(B).** Clonogenic survival assay for cisplatin sensitivity in MCF7 cells control and MCF7_RPA2_KD cells. **(C).** RPA2 siRNA construct 2 knock down in MDA-MB-231 cells. Lysates were collected at day3 and day5. (**D**) Clonogenic survival assay for cisplatin sensitivity in MDA-MB-231 cells control and MCF7_RPA2_KD cells. Statistical analysis was conducted as on GraphPad Prism7 software. To compare between two groups, student- T-tests analysis was performed. One-way ANOVA was performed to compare between more than two groups (variances analyses). Two-way ANOVA was used to analyse two variables such as Annexin V analysis and cell cycle analysis. All experiments were expressed as means ± standard deviation S.D. of three independent experiments. Error bars represent standard error of mean between experiments. UN = untreated, T = treated. '*' = p value <0.05, '**' = p value <0.001, '***' = p value <0.0001.

Uncropped gels

Supplementary Figure 17

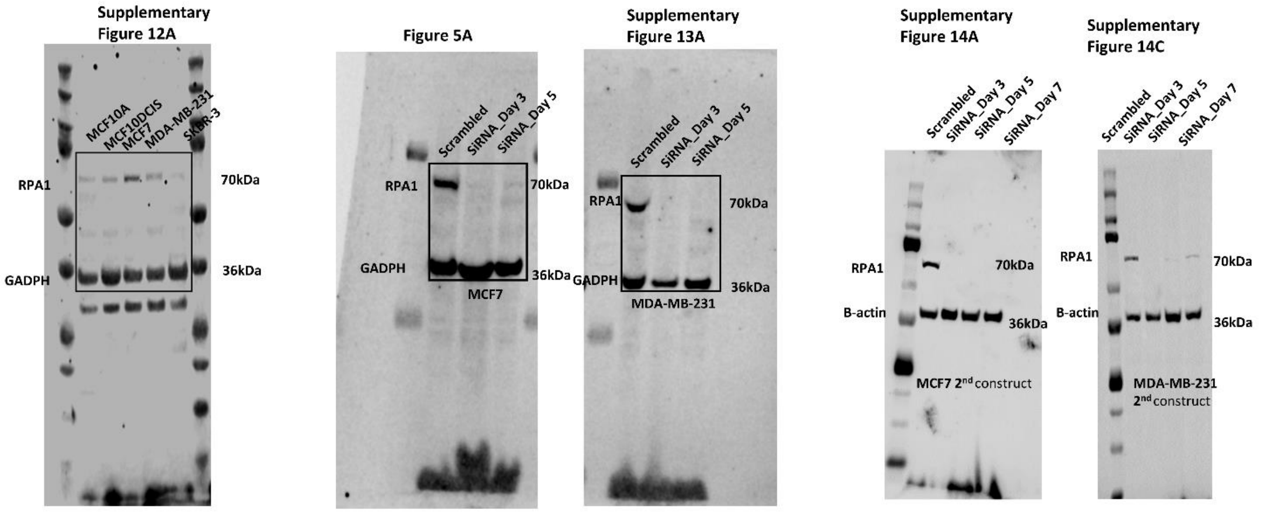

Supplementary Figure 18

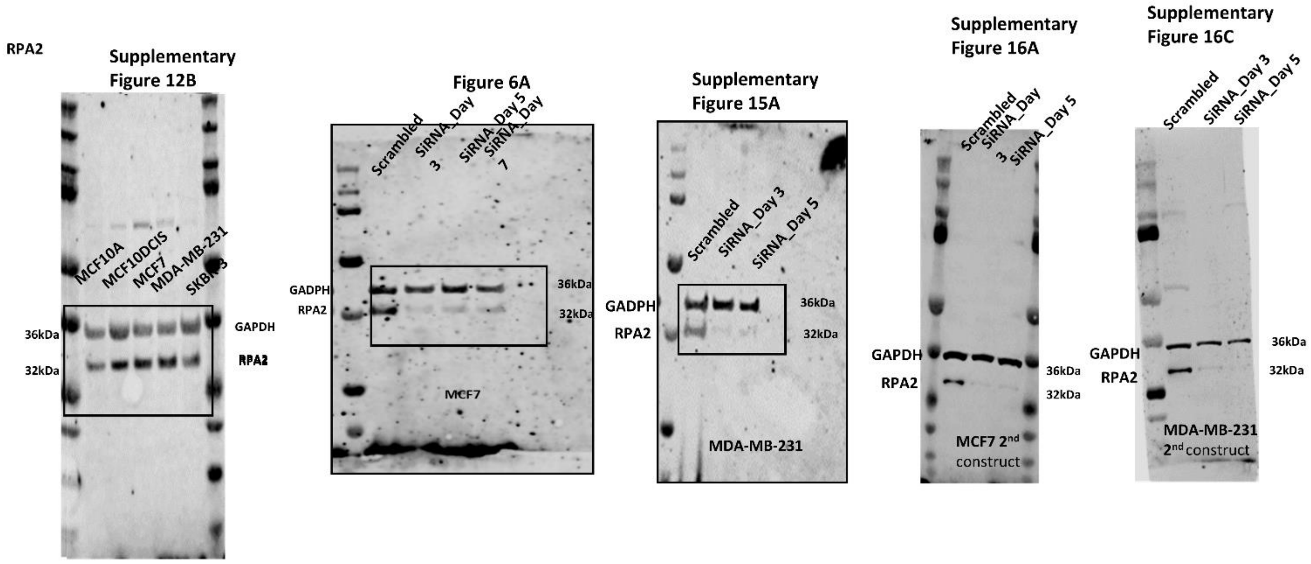

Supplementary Figure 19

Supplementary Figure 12E

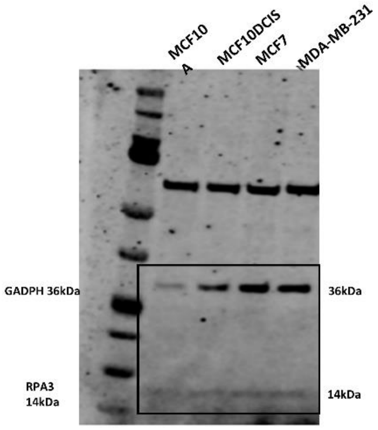

Supplement: Supplementary file 1 — Supplementary Tables_Figures_Figure legends [file 41523_2023_524_MOESM1_ESM.pdf]
